# Contrastive Machine Learning to Quantify Hypertensive Multiorgan Damage and Identify New Disease Phenotypes: A Multinational Multimodal Study

**DOI:** 10.1161/CIRCULATIONAHA.125.077394

**Published:** 2026-06-21

**Authors:** Mohanad Alkhodari, Winok Lapidaire, Turkay Kart, Zhaohan Xiong, Samuel Krasner, Andrew J. Fletcher, Shakila Bibi, Natalie Savage, Katie Suriano, Tobias R. Baumeister, Eric O. Ohuma, Ana I.L. Namburete, Pablo Lamata, Yasser Iturria-Medina, Lucy C. Chappell, Christina Y.L. Aye, Basky Thilaganathan, Abigail Fraser, Lucy Mackillop, Richard J. McManus, Ntobeko A.B. Ntusi, Ahsan H. Khandoker, Leontios J. Hadjileontiadis, Adam J. Lewandowski, Abhirup Banerjee, Paul Leeson

**Affiliations:** 1 Cardiovascular Clinical Research Facility (CCRF), Division of Cardiovascular Medicine, Radcliffe Department of Medicine, University of Oxford, United Kingdom (M.A., W.L., T.K., Z.X., S.K., A.J.F., S.B., N.S., K.S., A.J.L., P.L.).; 2 Healthcare Engineering Innovation Group (HEIG), Department of Biomedical Engineering & Biotechnology, Khalifa University, Abu Dhabi, United Arab Emirates (M.A., A.H.K., L.J.H.).; 3 Neuroinformatics for Personalized Medicine Lab, McGill University, Montreal, Canada (T.R.B., Y.I.-M.).; 4 London School of Hygiene and Tropical Medicine, University of London, United Kingdom (E.O.O.).; 5 Oxford Machine Learning in Neuroimaging Lab, Department of Computer Science, University of Oxford, United Kingdom (A.I.L.N.).; 6 School of Biomedical Engineering and Imaging Sciences, Kings College London, United Kingdom (P.L.).; 7 Department of Women and Children’s Health, Kings College London, United Kingdom (L.C.C.).; 8 Nuffield Department of Women’s and Reproductive Health, University of Oxford, United Kingdom (C.Y.L.A., L.M.).; 9 Molecular and Clinical Science Research Institute, St George’s University of London, United Kingdom (B.T).; 10 Bristol Medical School, University of Bristol, United Kingdom (A.F.).; 11 Brighton and Sussex Medical School, University of Brighton and University of Sussex, Brighton, United Kingdom (R.J.M.).; 12 South African Medical Research Council, Cape Town, South Africa (N.A.B.N.).; 13 Department of Medicine, University of Cape Town, South Africa (N.A.B.N.).; 14 Department of Electrical and Computer Engineering, Aristotle University of Thessaloniki, Greece (L.J.H.).; 15 Nuffield Department of Population Health, University of Oxford, United Kingdom (A.J.L.).; 16 Institute of Biomedical Engineering, Department of Engineering Science, University of Oxford, United Kingdom (A.B.).

**Keywords:** hypertension, machine learning, organ damage, progression trajectory, pseudotemporal modeling

## Abstract

**BACKGROUND:**

Hypertension induces structural and functional damage in multiple organs. Evidence of subclinical damage increases risk of vascular events and death but can be difficult to identify in the clinic. We developed a novel machine learning approach that quantifies current hypertension-associated multiorgan damage, mapping progression from health to advanced disease, in a pseudotemporal manner and predicts organ-specific disease progression trajectories.

**METHODS:**

We analyzed 566 multimodal imaging and nonimaging variables from 27 099 participants in the UK Biobank imaging substudy to develop a semisupervised contrastive trajectory inference (cTI) framework that models multiorgan alterations associated with hypertension exposure, including heart, brain, kidneys, vasculature, lungs, liver, and metabolic information. Model stability was validated through multiple internal validation steps, and external validity was tested on 5507 participants from the Atherosclerosis Risk in Communities study (ARIC). Clinical relevance was evaluated against existing risk scores and through ability to predict survival and incident multiorgan disease for up to 7 years, across both UK Biobank and ARIC.

**RESULTS:**

In the UK Biobank (mean age 63.27±7.48 years; 53.4% women) our global organ damage score (HyperScore) achieved an area under the curve of 0.964 (0.941–0.987) for identification of individuals with severe end-organ disease and robust stability in cross-validation with a mean root mean square error of 0.104±0.084. Survival odds differed significantly across HyperScore stages (*P*<0.001), whereas stratification by blood pressure was nonsignificant. We further revealed 6 hypertensive disease phenotypes (HyperTrajectory), characterized by predominant cardiac, lipoprotein, atherothrombosis, brain, cardiorenal, and liver features, respectively. External testing in ARIC confirmed stability of the model, with Jensen-Shannon distances as low as 0.10 for HyperScore distributions, without significant deviation in organ damage progression patterns (*P*>0.05) and consistent end-organ and outcome characteristics between ARIC and UK Biobank across HyperTrajectories.

**CONCLUSIONS:**

Machine learning–derived global organ damage scores are feasible in hypertension and enable identification of distinct hypertension-associated organ-disease phenotypes. New frameworks for hypertension assessment and monitoring using imaging to derive personalized risk assessment and phenotype-specific intervention may be achievable.

**Clinical Perspective**

**What Is New?**

- This is a multimodal multiorgan machine learning model that can both, in a pseudotemporal manner, quantify the current hypertension-associated end-organ damage as a score and predict the associated organ-specific disease progression trajectory with excellent performance.
- There are 6 newly discovered organ-specific phenotypes in response to hypertension predominantly characterized by cardiac, lipoprotein, atherothrombosis, brain, cardiorenal, and liver features.
- The comprehensive assessment of current multiorgan alterations in response to hypertension improves risk prediction more effectively than blood pressure and comparably to existing risk scoring techniques.

**What Are the Clinical Implications?**

- This model provides insights into hypertension-associated organ-specific alterations, which could deliver more personalized clinical management and enable more targeted interventions.
- Such insights could enable earlier identification of high-risk individuals who would otherwise be missed by blood pressure monitoring alone, supporting preventive treatment before irreversible organ damage develops.
- The discovery of distinct phenotypes in response to hypertension could prompt further research into tailored therapeutic approaches and direct follow-up strategies according to dominant organ systems affected and predicted clinical outcomes.

Hypertension is a leading risk factor for cardiovascular disease–related deaths, which account for one-third of worldwide mortality.^1^ Elevated blood pressure causes structural and functional changes in the heart, vasculature, and brain that predispose to vascular events.^2^ Notably, these alterations start to be observed in some individuals even with mild blood pressure elevations,^3^ whereas some will not develop end-organ changes despite prolonged exposure to hypertension.^4–6^ Therefore, this heterogeneity means that treatment based on blood pressure level alone may undertreat or overtreat at an individual patient level. Risk scores such as Framingham^7^ and QRISK3^8^ can provide additional information on risk of future cardiovascular events, but they do not give information on current disease state.

Including extent and type of damage across multiple end-organs in clinical decisions, such as blood pressure treatment thresholds or medication selection, would require a holistic understanding of how organ remodeling emerges and changes during the life course of hypertensive disease.^9–11^ Traditionally, longitudinal multimodal imaging data sets, acquired over decades, would be required to uncover these patterns.^12,13^ We hypothesized that applying a semisupervised contrastive machine learning approach to a sufficiently large cross-sectional imaging data set could address this challenge and, for the first time, reveal unique patterns of multiorgan changes related to hypertension.

Therefore, we developed a pseudotemporal contrastive trajectory inference (cTI) framework able to distinguish normotensive individuals from those with advanced hypertensive end-organ disease within the UK Biobank imaging data set. By positioning individuals within this framework, relative to the healthy state, we could derive a quantitative measure of current global end-organ disease severity (HyperScore). Additionally, clusters were evident within the contrastive space describing specific patterns of end-organ change (HyperTrajectories).

Clinical relevance was tested using prospective data to demonstrate that individuals with more severe end-organ disease, defined by a higher HyperScore, had events sooner than those with less advanced disease, and that the nature of the clinical event was consistent with the predominant organ changes defined by the HyperTrajectory. Finally, we tested stability of the model and reproducibility of the associations with clinical outcomes in imaging data from the Atherosclerosis Risk in the Community study (ARIC) that is both geographically distinct and ethnically diverse.

## Methods

### Data Set for Model Development

For model development, we used data from the Imaging Enhancement Study (2015–ongoing) of the UK Biobank.^14^ The study complies with the Declaration of Helsinki, was approved ethically by the Northwest Multi-Center Research Ethics Committee (MREC) as a Research Tissue Bank (RTB) approval (REC reference: 11/NW/0382), and received approval extension in 2021 (REC reference: 21/NW/0157). A written consent form was obtained from every participant before conducting the imaging study.

### Data Curation and Cohort Selection

#### Data Preparation and Feature Selection

We selected 27 099 participants from the UK Biobank who had available multimodal data, including blood pressure measurements (Expanded Methods). These individuals had no previous heart attack, angina, or stroke. We performed feature selection to reduce redundancy and enhance variability across different modalities. Briefly, the selection was based on reducing the overall covariance between variables per modality. Accordingly, 566 variables were included, collecting most of the complex relationships at every modality (Figure S1).

#### Adjusting Covariates and Harmonization

We adjusted for covariates using robust linear regression to reduce the effects of confounding factors (age and sex) that were not controlled during the imaging study. In addition, we used the recent ComBat tool^15^ to perform data harmonization across the 3 imaging centers where the imaging substudy was conducted. Harmonization was important to reduce the variance caused by nonbiological sources in multicenter studies.

#### Definition of Contrastive Groups

**Figure 1.**
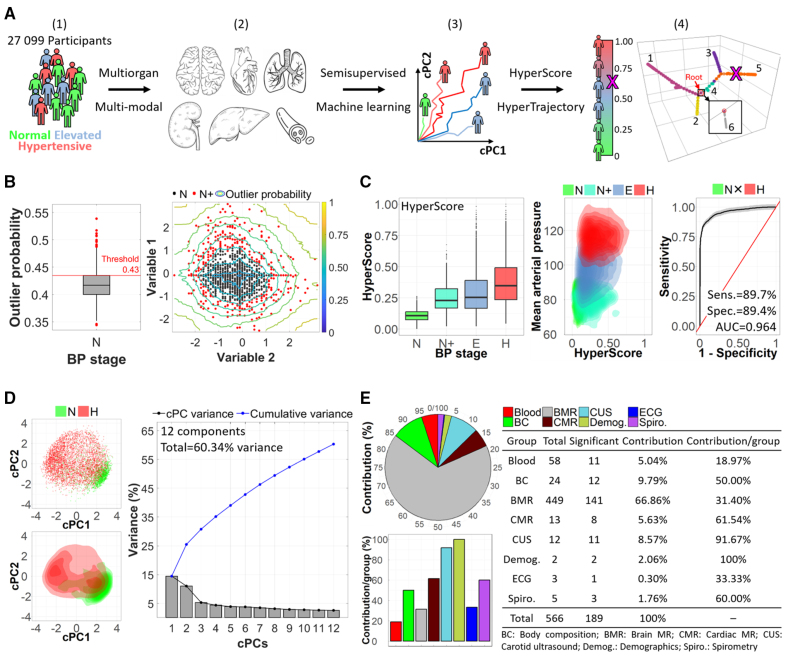
**Machine learning-based multiorgan and multimodality modeling of the end-organ changes associated with hypertension. A**, Overview of the modeling approach that starts by: enrollment of participants with different blood pressure stages at the UK Biobank imaging study (2015–ongoing; 1) to obtain variables of multiple organs using different imaging modalities and nonimaging measurements including brain magnetic resonance (BMR), cardiac MR (CMR), carotid ultrasound (CUS), electrocardiography (ECG), spirometry (Spiro.), body composition (BC), blood biochemistry, and alongside demographics (Demog.; 2). Then, data pass through a semisupervised contrastive trajectory inference (cTI) approach using contrastive principal component (cPC) analysis and minimum spanning tree (MST; 3), which aid in assigning a numerical value estimating the disease-state risk for any individual, HyperScore, and locating the individual on a constructed disease pathways map, HyperTrajectory (4). **B**, Process of identifying outliers (N+) in the normal (N) group who are above the third quartile of probabilities (≥0.43) assigned using isolation forest and clinical variables. **C**, Estimating HyperScore and grouping patients relative to their blood pressure (BP) level and stage, (ie, N, N+, intermediate elevated [E], and hypertensive [H]). The discrimination between N and H showed an excellent (96.4%) area under the receiver operating characteristic (AUC) curve, with a sensitivity (Sens.) and specificity (Spec.) of 89.7% and 89.4%, respectively. **D**, Interpretation of cPCA modeling for the N and H groups in the cPC1 and cPC2 space, with the total variance (60.34%) explained by the selected 12 components. **E**, Importance of clinical variables relative to the modeling grouped by modality.

The proposed study requires contrastive modeling using participants considered the “healthiest” and comparing these with participants likely to be representative of advanced disease state. The “healthy” state includes individuals who would not be expected to have evidence of hypertensive end-organ damage, and we refer to these as normotensive “N.” We defined this group as participants having systolic blood pressure (SBP)/diastolic blood pressure (DBP) <120/80 mm Hg during their first imaging visit. In addition, they had never received any blood pressure medications and had never been diagnosed with hypertension. This group was further refined using an isolation forest^16^ applied to imaging features to filter outlier individuals with imaging characteristics suggestive of nonhypertensive pathology or remodeling, the “N+” group (Figure 1B; Expanded Methods).

We then identified the group most likely to have evidence of end-organ disease and referred to it as the hypertensive “H.” These participants had either an SBP or DBP of >160 or 100 mm Hg, respectively, in line with stage 2 hypertension as defined by the 2014 Eighth Joint National Committee (JNC 8) guidelines,^17^ 2017 American Heart Association/American College of Cardiology (ACC/AHA) guidelines,^18^ 2023 European Society of Hypertension (ESH) guidelines.^19^ All remaining participants were assigned to an intermediate elevated group “E.” The E group was not involved in model development but was subsequently used during validation, discovery and analysis.

### Model Development

#### Contrastive Modeling

To capture the most informative contrastive components in the data, reduce data set dimensionality, and develop a model that incorporates joint information across multiorgan data, we used the contrastive principal component analysis (cPCA) algorithm.^20^ Contrary to the conventional PCA algorithm, cPCA performs weighted subtraction between individual covariance matrices of the 2 extremities (ie, group N and group H), to form the contrastive covariance matrix (Figure S2).

Upon obtaining the reduced dimensional cPCA space, we performed cTI^21^ by first calculating the Euclidean distance matrix of the cPCA components among all participants from groups N and H. The healthiest participant, labeled as the “root node,” was automatically selected based on identification of the closest participant to all other N participants. The root node was then used to create a minimum spanning tree (MST)^22^ undirected graph with the aim of forming optimal connections that minimize the summed length of all paths from the root node. The distance of each participant from the root node was generated within a normalized range (0–1) relative to the maximum value (the farthest distance from the root node) that should represent the extreme end-organ condition. This value provides a quantitative measure of end-organ disease state (ie, the proximity of each participant from the pathology-free state relative to the advanced disease state).

#### Clustering Graph Connections

As participants were not distributed homogenously through the cPCA-MST space, we also investigated whether there were dominant “clusters” of participants tracking through the space from the root node to various advanced disease states (ie, “trajectories” of end-organ change). To do this, we identified dominant contrasted pseudotemporal connections in the MST. We used Laplacian transformation^23^ to create an embedding Eigenspace with no dimensional meaning for the axes. We then defined major pathways in the constructed graph using a systematic approach that reduced the overall overlap between clusters (Figure S3; Expanded Methods). We targeted that each major cluster (ie, “trajectory”) included at least 10% of the participants; a threshold that we chose a priori to avoid bias in trajectory definition. After this step, 6 major “trajectories” were definable.

### HyperScore and HyperTrajectory Definitions

For ease of description, we refer to the distance of each participant from the root node (ie, the quantitative measure of end-organ disease state) as their HyperScore. Similarly, we also describe the “trajectory” to which each participant was allocated as their HyperTrajectory. To simplify group comparison, we also allocated participants to 3 HyperScore stages, namely low, medium, and high (Expanded Methods). We developed a methodology to assign a HyperScore and identify the most likely HyperTrajectory for participants who were not included in the development of the baseline model (eg, in testing sets). To estimate a HyperScore and HyperTrajectory with probabilities, we used a *k*-nearest neighbour (*k*-NN) approach^24,25^ of *k*=25 to find the similar participants in the original data set to the hidden testing data (Expanded Methods).

### Internal Validation

#### Stability of HyperScores Generation

We performed multiple internal validation scenarios by building models based on smaller subsets of the full data set. We then used these models to predict HyperScores for participants in the held-out data. The regenerated HyperScores were compared with the scores generated originally when modeling with the full data set, and size of differences between scores was evaluated using root mean square error (RMSE), with an expectation that there would be an ≈10% variation between scores. We did this in 2 ways. First, we used a stratified 10-fold cross-validation scheme. Second, we built 2 separate models based on half the available data. We did this by splitting the data randomly into 2 data sets representing 50% of the participating cohort and by splitting the data set into 2 separate models based on the time of the imaging visit, namely from 2015 to 2018 and from 2018 to 2021. Normalization and data imputation were performed on each subset separately before modeling to ensure no data leakage. We further performed analysis in the absence of ground-truth,^26^ analysis of data drifting, and analysis of Jensen-Shannon (JS) divergence^27,28^ (Expanded Methods).

#### Stability of HyperTrajectory Allocation

We performed internal validation with stratified 10-fold cross-validation to evaluate the regeneration of the HyperTrajectory (Expanded Methods). We used the MST graph created on each fold and re-generated 6 major pathways within that data. Accordingly, each individual within the held-out data was then attributed a probability for location on each trajectory. This allocation was compared to their original attribution within the full model. We used performance analysis using top 2 most likely trajectories (ie, a prediction is considered correct if it was predicted among the highest 2 predicted trajectories).

### Clinical Relevance of HyperScore

We first visually assessed how individual clinical phenotypes known to vary with exposure to hypertension varied across the HyperScore ranges. We then hypothesized that individuals with more advanced end-organ changes should be closer to having a hypertensive-related event than individuals who may have similar blood pressure but have less evidence of end-organ disease. To test this hypothesis, we used prospective follow-up data and studied time-to-event within each HyperScore range using Kaplan-Meier^29^ testing with a significance threshold of 0.05. The diagnosis was derived from the *International Classification of Diseases*, 10th revision (*ICD-10*) data (Expanded Methods) and analyzed as an event-free probability. Additionally, to evaluate independence from blood pressure level, we studied the time-to-event with the study group stratified by blood pressure level in 2 ways, namely 4 groups and 9 groups (Expanded Methods).

As a final evaluation of clinical relevance, we compared how effectively HyperScore predicts time-to-event versus widely available “gold standard” risk models, optimized to predict future events (eg, QRISK3,^8^ ACC/AHA,^30^ Framingham,^7^ and MESA [Multi-Ethnic Study of Atherosclerosis]),^31^ as well as an imaging measure proposed as predictive risk marker (ie, carotid intima-media thickness [IMT]). We further fitted multivariable Cox models^32^ incorporating the score, age, and sex and reported different performance metrics for comparison (Expanded Methods). Moreover, we assessed the ability of HyperScore to predict longer term outcomes using a fixed time horizon for events of 4 years or longer after the imaging visit and compared its performance with that of other metrics (Expanded Methods).

### Clinical Relevance of HyperTrajectory

To study whether the HyperTrajectory provided additional biological and clinical relevance beyond that obtained from the HyperScore alone, we examined demographic information, disease outcomes, time-to-event, and imaging phenotypes across the 6 HyperTrajectories. We summarized the trajectories by identifying distinct patterns evident across them. Here, following a systematic approach, we allocated to each HyperTrajectory the predominant characteristic evident (ie, the major end-organ–related attribute that had the most marked differences at the highest 5% HyperScores within that HyperTrajectory), which represents the advanced disease state (Expanded Methods).

### External Testing

#### External Testing Data Set

We sought to formally test, on an independent external data set, the reproducibility of the clinical relevance of HyperScore and HyperTrajectory observed in the internal validation steps. We used data collected within the ARIC study.^33,34^ All 4 clinical centers where the study was conducted approved ARIC protocols through their institutional review boards, and participants provided written consent before the enrollment. We focused on participants in visit 5 (ie, between 2011 and 2013), as this visit involved brain magnetic resonance (MR) examinations (Expanded Methods).

#### External Testing of Clinical Relevance

We first generated a HyperScore and probability of allocation to HyperTrajectory for all participants in the ARIC data set using normalized variables to account for variation in normal ranges between studies. HyperScores and HyperTrajectories were predicted based on available variables per participant, for which the corresponding cPC coefficients were selected from the overall cPCA space to apply the transformation. We then performed the exact same protocol for evaluation of clinical relevance as used in internal clinical validation. This included visual assessment of the change in individual phenotypes with increasing HyperScore and study of time-to-event according to HyperScore range. We then looked at HyperTrajectory allocation and studied variation in individual phenotypes and time-to-event. All findings were compared to those observed in internal validation to evaluate the consistency of findings. Finally, we performed preliminary intersectional analyses by sex and ethnicity and reported observations related to HyperScore and HyperTrajectory in both data sets.

### Statistical Methods and Regression Analysis

All statistical tests were carried out using the 2-sided analysis of variance (ANOVA) with a significance level of 0.05. To evaluate phenotypic patterns, we performed regression analysis using locally estimated scatterplot smoothing (LOESS) fitting and compared it with linear fitting. Error percentage was provided as the overall ratio of RMSE between data points and the fitted line. Linearity analysis was performed by evaluating the coefficient of determination (*r*^2^) provided by the fitted line to the data points. Moreover, the *P* value shows the significance of this fitting relationship. We performed statistical analysis over the fitted line coefficients (slope) to compare models during the validation of phenotypic patterns. The statistical analysis of variables predicting trajectories was performed using a linear mixed effects model adjusted for age and sex. Pairwise comparisons between trajectory groups were performed using least-squares means, with *P* values adjusted using the Bonferroni method to control the family-wise error rate.

## Results

### Study Population

The baseline data set included 14 467 women (53%) and 12 632 men (47%), with a mean age of 63.3±7.5 years, and the majority were from a White ethnic background (26 254; 97%). Of these participants, 3450 (13%) had pre-existing hypertension before their imaging visit, 5823 (21%) participants were taking antihypertensive agents, and 5563 (21%) had cholesterol-lowering medications. Detailed characteristics are provided in the Table and Table S1.

**Table.**
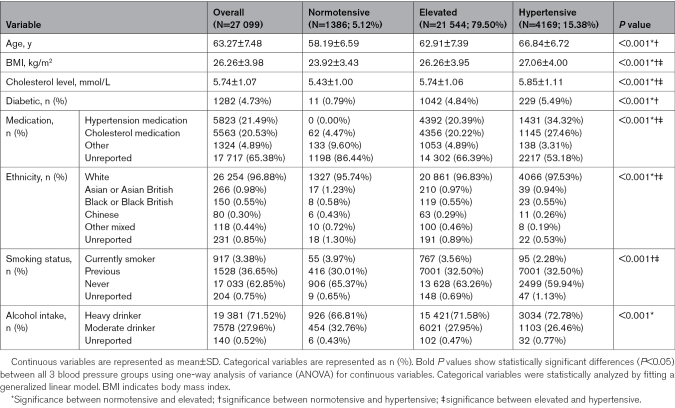
Baseline Characteristics of the UK Biobank Cohort Included in This Study.

### Modeling Analysis

A total of 1386 participants (5.12%) were referred to as the “N” group; 4169 (15.38%) were deemed likely to have significant hypertensive end-organ changes (ie, the “H” group). This left 21 544 individuals (79.50%) grouped within intermediate groups. The developed model was able to discriminate between the estimated HyperScores within the N and H groups with an area under the curve (AUC) of 0.964 (0.941–0.987; *P* for trend <0.001; Figure 1C), indicating good separation of these 2 disease states within the model. Figure 1D shows the effective differentiation of H and N groups, using the first 2 cPCs for illustration purposes. Twelve cPCs captured the majority of the contrastive differences, explaining 60.3% of the overall variance between groups. When comparing the N and H groups, 189 variables were associated with group membership at *P*<0.05 (Figure 1C), in which the top 3 major contributions came from the brain MR biomarkers (66.9%), body composition measures (9.8%), and carotid ultrasound (8.6%). The top 15 measurements that contributed the most to the modeling (Figure S4) included white matter hyperintensities (7.29%), trunk fat mass (1.67%), and mean carotid IMT (1.37%).

### Internal Validation

#### Stability of HyperScore Estimation

**Figure 2.**
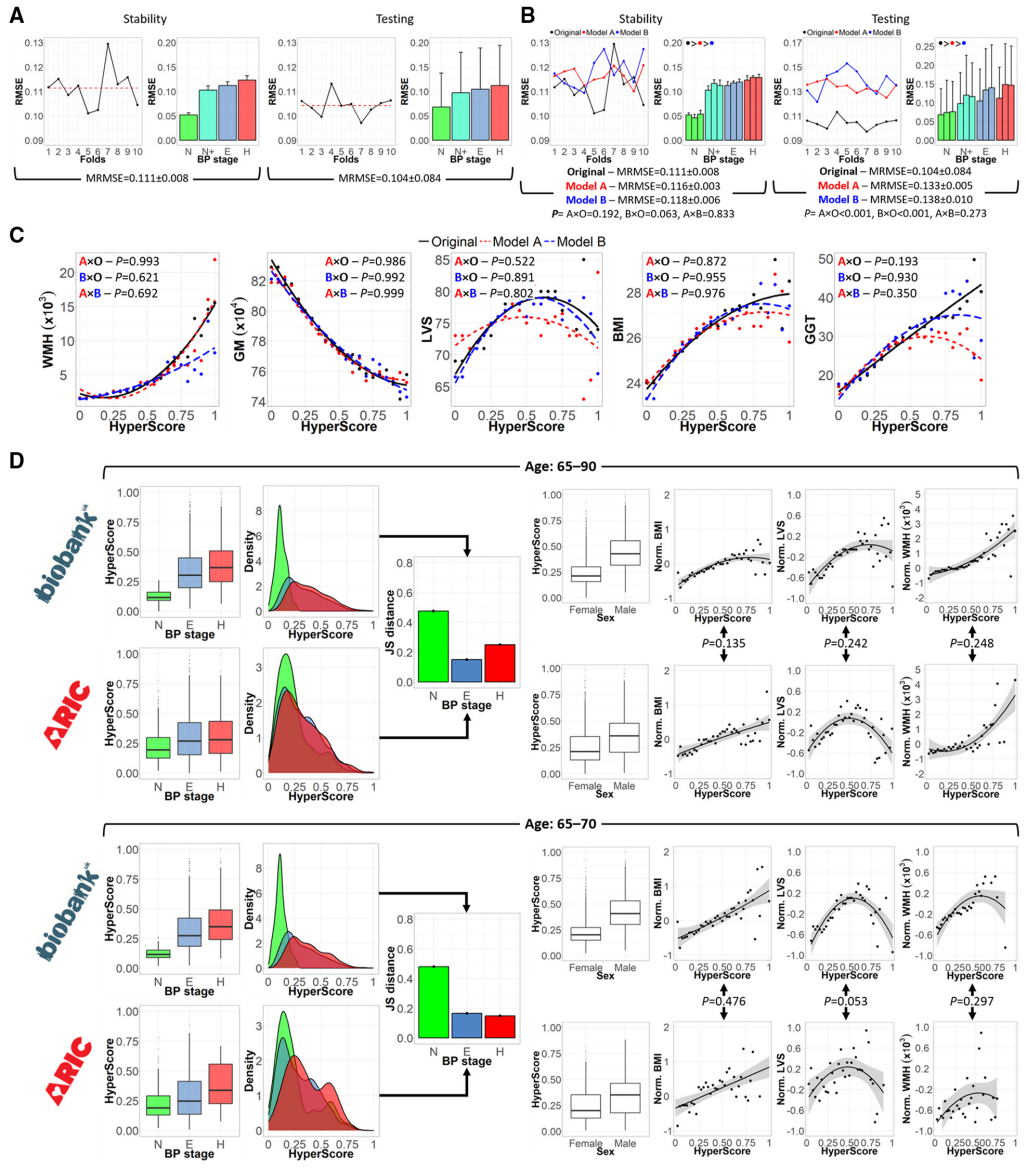
**Internal and external validation of the modeling approach and HyperScore estimation performance. A**, Evaluating model stability and testing in predicting HyperScore when changing data used to generate the model. A stratified 10-fold cross-validation scheme was followed, and difference in HyperScore between the full data set and per-fold subsets was calculated using the mean root mean square error (RMSE) to find stability. Model error in predicting HyperScore for testing subsets at each fold was calculated to find testing performance as well. **B**, Comparing stability and testing performance when generating 2 additional models (**A** and **B**) using 50% (n=13 550) of the overall participants cohort in each. **C**, Calculating the statistical difference between the 3 models (original, **A**, and **B**) for several phenotypic patterns. Nonsignificance indicates similar phenotypic patterns across the models. **D**, External validation on the Atherosclerosis Risk in Communities study (ARIC) subset of 5507 participants for 2 age groups, namely 65–90 years (overall cohort) and 65–70 years (younger subset), and participants from the UK Biobank were selected according to the same ranges. HyperScore distribution at every blood pressure (BP) stage was compared using Jensen-Shannon distance calculations. Phenotypical patterns (*z*-score normalized values) with respect to HyperScore for the ARIC subsets were provided alongside their counterparts from the UK Biobank and were statistically compared based on slope. Points within plots represent median value of participants at each 0.025 HyperScore step. BMI indicates body mass index; GGT, gamma-glutamyl transferase; GM, grey matter; JS, Jensen-Shannon; LVES, left ventricular stroke volume; MRMSE, mean RMSE; and WMH, white matter hyperintensity.

The stratified 10-fold cross-validation showed no significant differences between scores across folds (Figure 2A) based on a priori acceptance criteria, with a mean RMSE (MRMSE) of 0.11±0.01. Moreover, the predictability of testing sets at each fold had an MRMSE of 0.10±0.08. In both analyses, there was an association between the absolute level of HyperScore for an individual and the variance in their HyperScore across folds, with higher HyperScores having wider variance. More validation details are provided in the Extended Results.

#### Stability of HyperTrajectory Allocation

The stability of the HyperTrajectory regeneration demonstrated the highest stability for trajectories 1 and 5, with a sensitivity of 64±5.8% and 74±6.8%, respectively (Figure S6). The highest precision values were achieved by trajectories 1 and 2, reaching up to 68.96±3.85% and 61.98±13.80%, respectively. On the other hand, trajectory 6 could not be consistently reproduced on cross-fold testing. This trajectory had the smallest number of participants with a low average HyperScore, suggesting minimal variation in end-organ change. Similarly, the prediction of the testing set across folds using the regenerated HyperTrajectory demonstrated that trajectories 1 and 5 had the highest sensitivity measures at 64±7.6% and 79±7.2%, respectively, whereas trajectory 6 again could not be identified reproducibly.

### Clinical Relevance of HyperScore

#### Association With Demographics and End-Organ Phenotypes

**Figure 3.**
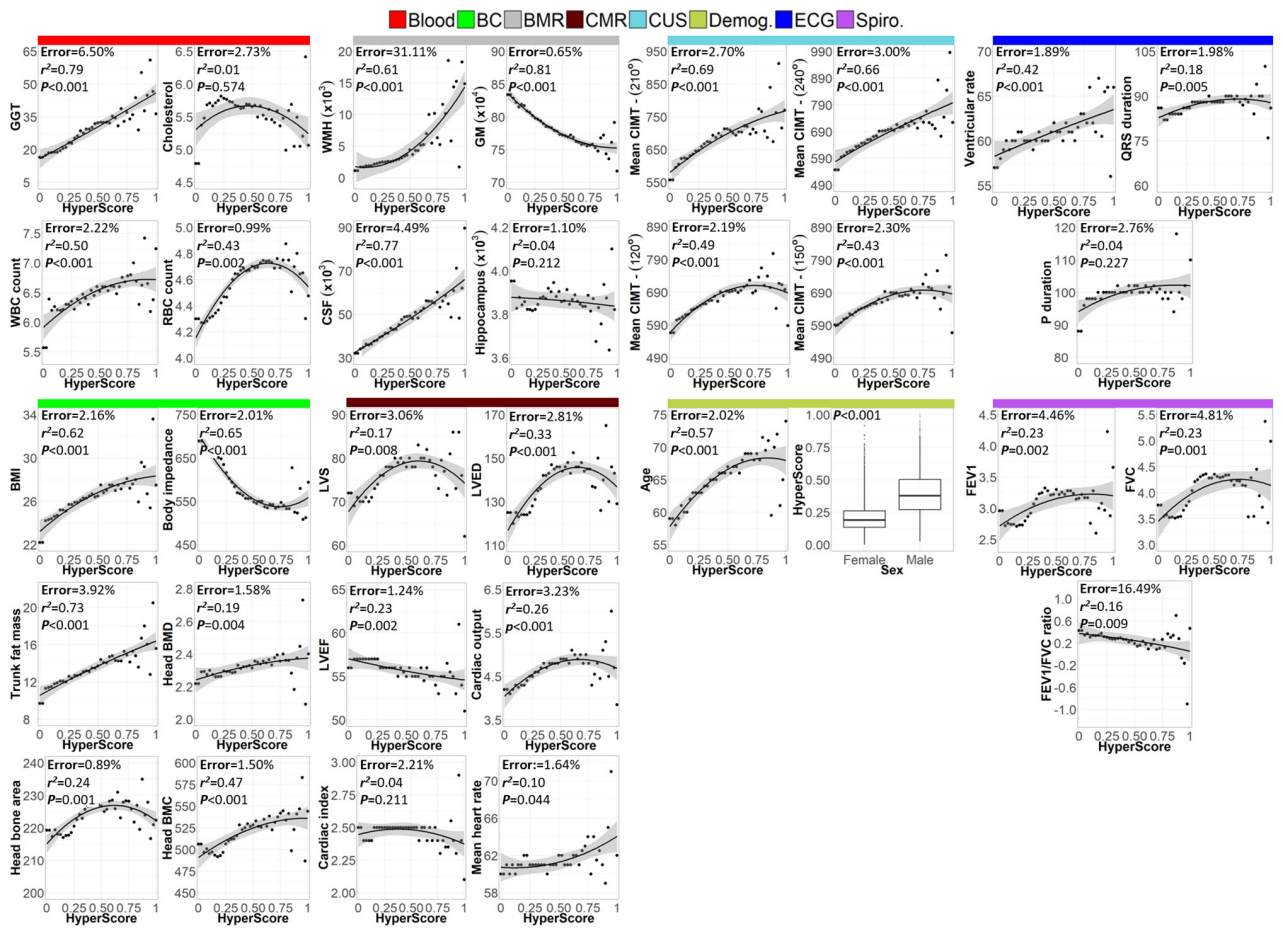
**Association between end-organ phenotypes and hypertension progression.** Phenotypic patterns with respect to HyperScore showing examples from every modality. Percentage error was calculated between the fitted line using locally estimated scatterplot smoothing (LOESS) and points, whereas the *r*^2^ and *P* value were obtained by fitting a linear model. Low *r*^2^ and nonsignificant *P* value indicate that there is no linear relation. Points within plots represent median value of participants at each 0.025 HyperScore step. BC indicates body composition; BMC, bone mineral content; BMD, bone mineral density; BMI, body mass index; BMR, brain magnetic resonance; CIMT, carotid intima-media thickness; CMR, cardiac MR; CSF, cerebrospinal fluid; CUS, carotid ultrasound, ECG, electrocardiography; FEV1, forced expiratory volume in 1 second; FVC, forced vital capacity; GGT, gamma-glutamyl transferase; GM, grey matter; LVED, left ventricular end-diastolic volume; LVEF, left ventricular ejection fraction; LVS, left ventricular stroke volume; MR, magnetic resonance; RBC, red blood cell; Spiro., spirometry; WBC, white blood cell; and WMH, white matter hyperintensities.

We found that men had a higher estimated HyperScore than women (*P*<0.001; Figure 3). Similarly, in regression analysis, a higher HyperScore was associated with more white matter hyperintensities (*r*^2^=0.61; *P*<0.001) and less grey matter volume (*r*^2^=0.81; *P*<0.001). Moreover, a positive association was observed with body mass index (BMI; *r*^2^=0.62; *P*<0.001), mean carotid IMT (*r*^2^=0.69; *P*<0.001), ventricular rate (*r*^2^=0.42; *P*<0.001), gamma-glutamyl transferase (GGT; *r*^2^=0.79; *P*<0.001), and age (*r*^2^=0.57; *P*<0.001), whereas a negative association was observed for body impedance (*r*^2^=0.65; *P*<0.001). A nonlinear relationship was observed with both left ventricular stroke (LVS) volume and left ventricular end-diastolic (LVED) volume with an initial positive relationship, with an inflection point and a switch to an inverse relationship at high HyperScores consistent with expected pathological change.

#### Time-to-Event Analysis

**Figure 4.**
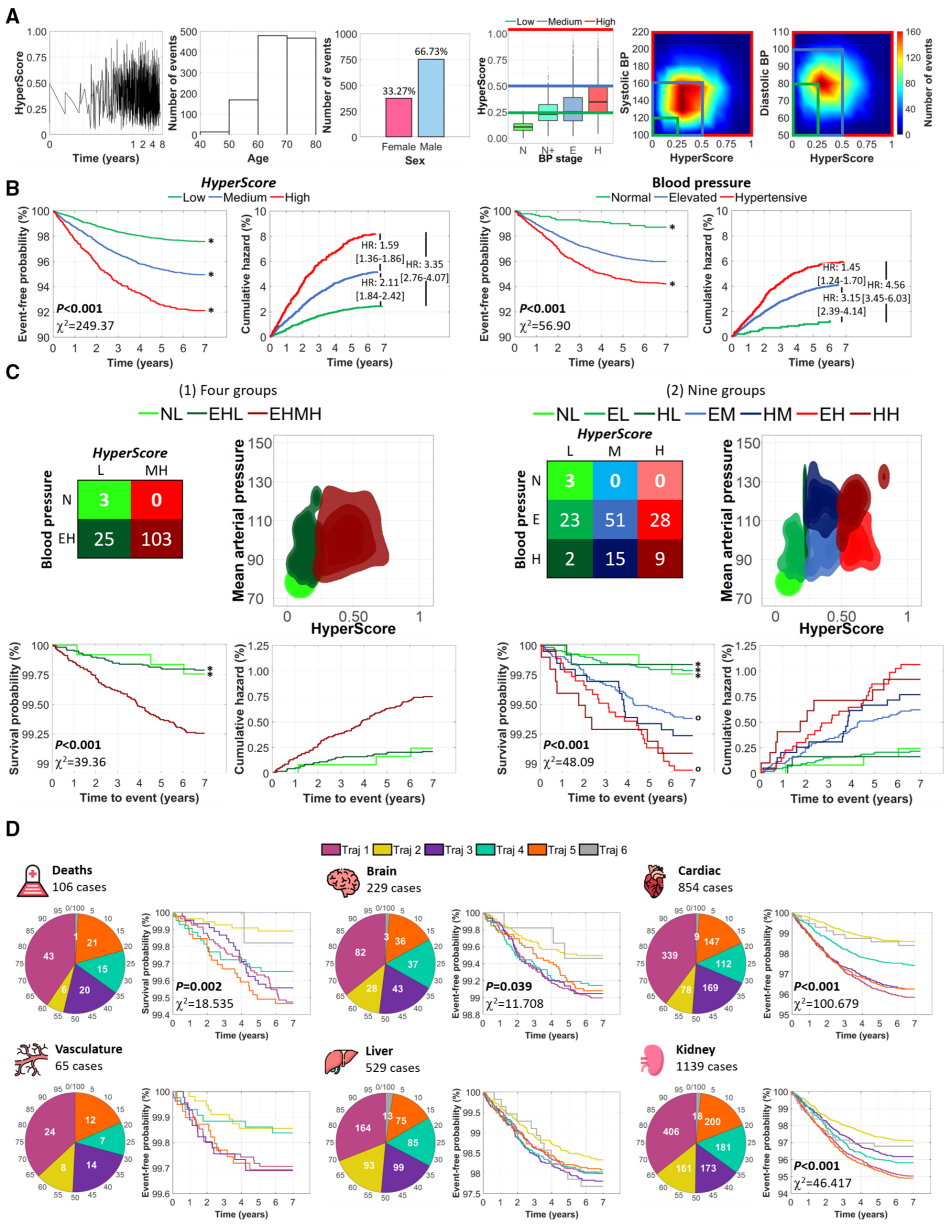
**Prediction of adverse outcomes and analysis of survival. A**, Overall representation of the 1127 participants who experienced events (circulatory deaths; *International Classification of Disease*, 10th revision [*ICD-10*]; circulatory diseases) with stratified analysis based on HyperScore stages. **B**, Analysis of survival probability and cumulative hazard for these participants when grouped based on HyperScore stages (low, medium, and high) and blood pressure (BP) stages (normal, elevated, and hypertensive). *Significance between curves. **C**, Analysis when grouping participants into 4 (1) and 9 (2) groups based on combined blood pressure and HyperScore stages as normal (N) or low (L), elevated (E) or medium (M), and hypertensive (H) or high (H). **D**, Analysis of survival in HyperTrajectory (defined in Figure 5) with respect to death and multiple body organ diseases defined according to *ICD-10*–coded outcomes (Supplemental Material) with the total number of cases in every trajectory (Traj). The *P* value is reported only for significant curves and reflects overall statistical significance. HR indicates hazard ratio.

We identified a total of 1127 participants (375 women [33.27%] and 752 men [66.73%]) who had experienced adverse events related to circulatory system diseases (ie, cardiac, cerebral, or vascular disorders; n=1021) or died because of circulatory system diseases (n=106) since they had attended their first imaging visit (Figure 4A). Time-to-event analysis and cumulative hazard estimation confirmed a higher HyperScore associated with a shorter time to first event (Figure 4B). Those with high blood pressure showed significantly shorter time-to-event compared to the low blood pressure group, while there were significant differences in time-to-event between the high HyperScore group and both the low (*P*<0.001; *c*^2^=249.37) and medium (*P*=0.001; *c*^2^=56.90) HyperScore groups.

When we divided participants into 4 groups taking into account both their HyperScore (low or medium/high) and blood pressure level (normal or elevated/hypertensive; Figure 4C), we found that for those with elevated/hypertensive blood pressures, HyperScore contributed additional stratification with a significant difference in odds of time-to-event (*P*<0.001) observed between participants with low HyperScore compared with participants with medium/high HyperScore at each blood pressure stage. Repeating this analysis, but with participants divided into 9 groups, confirmed these findings, with medium and high HyperScore having progressively shorter time-to-event compared with those with low HyperScore at each blood pressure stage. Overall, we observed significance driven by HyperScore groups (horizontal coloring direction) as opposed to blood pressure stages (vertical coloring direction).

#### Comparison With Conventional Prediction-Based Tools

Comparison of our machine learning–based and imaging-based metric of current end-organ disease state, as a predictor of time-to-event, with clinical scoring systems confirmed that HyperScore had comparable predictive ability for future events (Figure S7), particularly circulatory deaths. In addition, HyperScore effectively predicted time-to-event for a range of other individual clinical outcomes relevant to hypertension, whereas performance of established risk scores and carotid IMT was less consistent across conditions. Multivariable Cox models confirmed that HyperScore had high performance for circulatory outcomes with ΔC-statistic and net reclassification index (NRI) as high as +0.024 and 18.2, respectively, outperforming blood pressure (Tables S9) and performing comparably to other established scoring systems (Tables S10). For other outcomes (ie, brain and deaths), HyperScore outperformed the majority of other scoring metrics. In addition, HyperScore remained strongly associated with outcomes and performed relatively similar to other scoring metrics when analysis was limited to longer-term outcomes occurring ≥4 years after assessment (Figure S8), whereas performance of carotid IMT lost significance.

### Clinical Relevance of HyperTrajectory Allocation

#### Variation in Demographics and End-Organ Phenotypes

**Figure 5.**
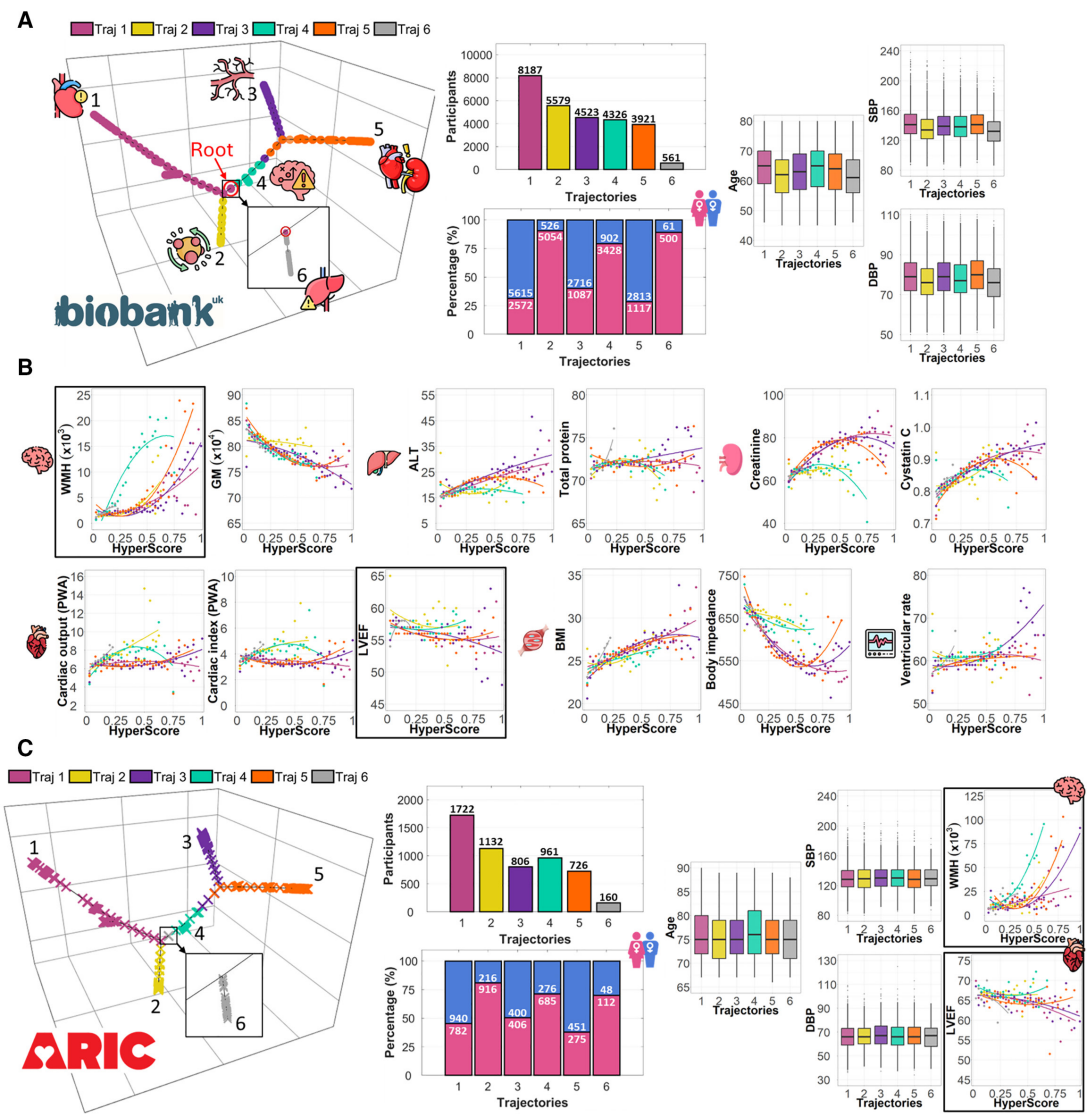
**Characterization of the HyperTrajectory in the UK Biobank and ARIC cohorts. A**, The trajectory map was created using the minimum spanning tree (MST) and visualized in the embedded Laplacian Eigenspace. Six major trajectories were formed after a systematic approach, and their demographics were analyzed including sex, age, and blood pressure. Each trajectory (Traj) was characterized by the increasingly occurring outcomes and organ-specific survival rate. These trajectories were characterized by predominant cardiac, lipoprotein, atherothrombosis, brain, cardiorenal, and liver features, respectively. **B**, Progression phenotypes in the HyperTrajectory. Each progression phenotype illustrates the change in the per-organ function with respect to HyperScore (ie, throughout the trajectory path). Points within plots represent median value of participants at each 0.025 HyperScore step. **C**, Placing each participant from the ARIC study (Atherosclerosis Risk in Communities) data set within the HyperTrajectory and observing their overall representation based on sex, age, blood pressure, progression phenotypes. More details on the dominant organ-based phenotypes appearing in **A** are provided in Figure 6. The black border around line plots is used for comparison between the UK Biobank and ARIC in terms of per-trajectory progression patterns for WMH and LVEF. ALT indicates alanine aminotransferase; BMI, body mass index; BP, blood pressure; DBP, diastolic blood pressure; GM, grey matter; LVEF, left ventricular ejection fraction; PWA, pulse wave analysis; and SBP, systolic blood pressure; and WMH, white matter hyperintensity.

Individuals within the 6 trajectories had similar median ages (Figure 5A and 5B). Three trajectories, namely trajectories 2, 4, and 6, were predominantly women (>79%), whereas the other 3 trajectories were comprised of relatively more men. There were no significant differences in mean SBP, DBP, pulse, or mean arterial pressure across the 6 trajectories. Visual inspection of the relative position and branching of HyperTrajectories, displayed as a 3-dimensional representation of the 6 trajectories, identified that trajectories 3 and 5 share a common pathway at lower levels of HyperScore and then separate as score increases. As expected, visual comparison of individual imaging biomarkers showed similar levels at the start of the HyperTrajectories, with divergence of phenotypes between HyperTrajectories with increasing distance from the root node. This was particularly evident for white matter hyperintensities, which demonstrated a gradual increase in the male-dominant trajectories 1, 3, and 5, and a much more accelerated increase in female-dominant trajectories 4 and 6.

Trajectory 1 had the lowest cardiac index and cardiac output during pulse wave analysis (PWA), reduced left ventricular ejection fraction (LVEF) on imaging, and the highest levels of creatinine, cystatin C, glucose, and glycated hemoglobin (HbA1c). Participants in trajectory 2 had relatively normal imaging-based health profiles but high levels of low-density lipoprotein (LDL), high-density lipoprotein (HDL), and total cholesterol. Trajectory 3 had an inflammatory profile of elevated white blood cells and reticulocytes. Trajectory 4 had high white matter hyperintensities and low grey matter volume. Trajectory 5 had low cardiac index, high creatinine, and high cystatin C. Trajectory 6 appeared to be related predominantly to liver-related outcomes but had a very limited sample size, therefore we are cautious about interpretation of this trajectory until larger sample sizes are available. Details are provided in Table S2 and Table S3 for the overall cohort and for the advanced disease state (ie, those individuals at the “tips” of the trajectories, respectively).

#### Variation in Events and Time-to-Event Across HyperTrajectories

**Figure 6.**
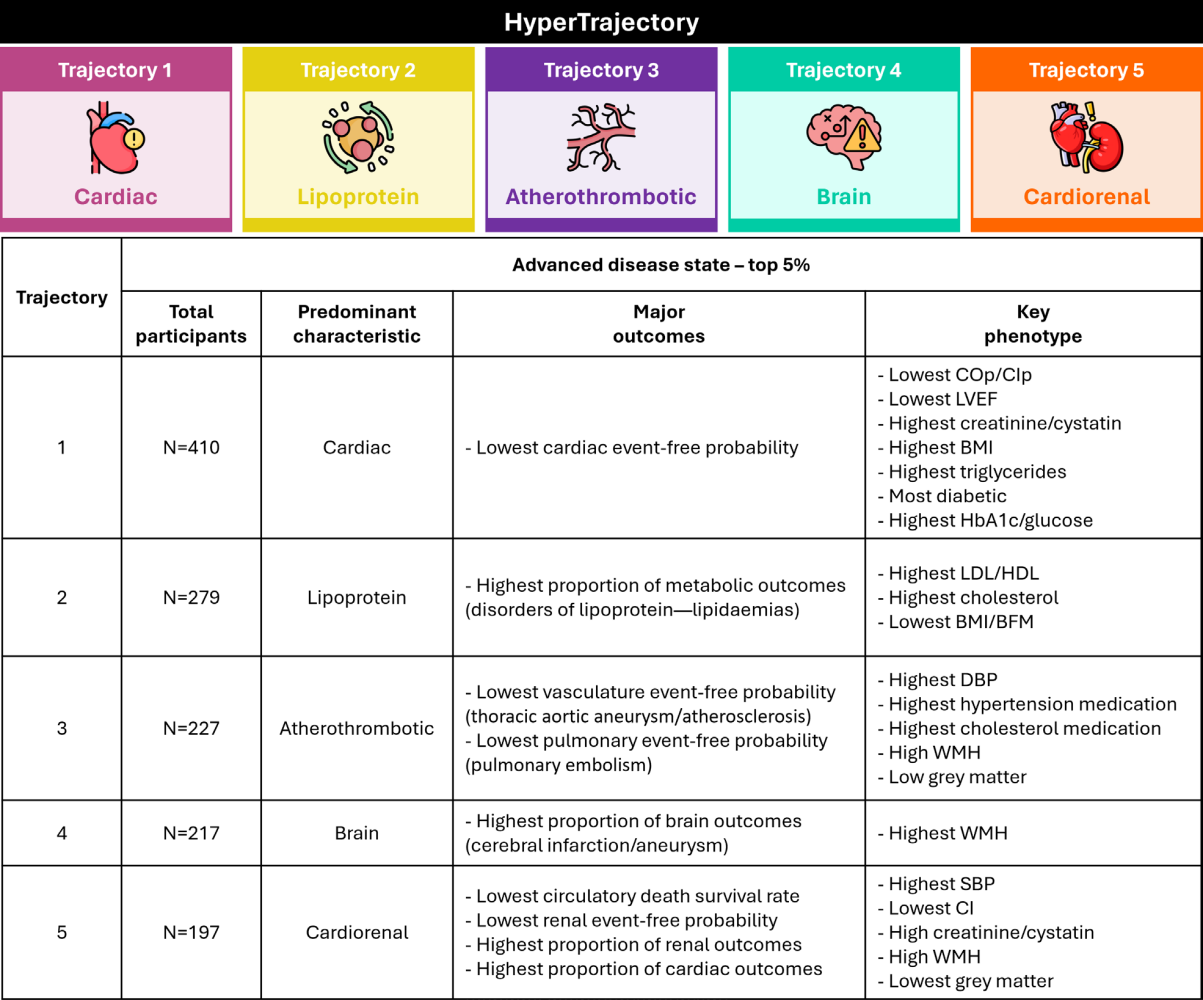
**Overall summary of the formed HyperTrajectories in the UK Biobank highlighting their HyperScore range, major characteristics, phenotype, and outcomes.** Participants were assessed based on their advanced disease state (highest 5% of HyperScores) at each trajectory. Predominant characteristics were derived from Table S3 as well as survival and time-to-event analysis. Trajectory 6 was not included in the summary because of having a low number of participants. BFM indicates body fat mass; BMI, body mass index; CIp, cardiac index; COp, cardiac output; DBP, diastolic blood pressure; HbA1c, glycated hemoglobin; HDL, high-density lipoprotein; LDL, low-density lipoprotein; LVEF, left ventricular ejection fraction; PWA, pulse wave analysis; RTIC, reticulocytes count; SBP, systolic blood pressure; WBC, white blood cell; and WMH, white matter hyperintensity.

Analysis of future outcomes for individuals within each HyperTrajectory (Figure 4D; Figure S9) showed striking similarities between disease outcomes and phenotype changes in each HyperTrajectory. Although all trajectories had a range of events, individuals within trajectory 1, who had phenotypic changes in cardiac measures, had the highest proportion of cardiac outcomes relative to other trajectories. Participants in trajectory 2, who were identified with changes in cholesterol level, had significantly lower mortality relative to all other trajectories (*P*<0.001; *c*^2^>10) but higher rates for most nonmortality events, with the highest proportion of metabolic outcomes, mostly lipoprotein disorders. Individuals in trajectory 3, who had changes in inflammatory markers, had relatively more vascular events including peripheral vascular disease and pulmonary thromboembolic disease. Trajectory 4, which had high white matter changes, had the highest proportion of brain disorders. Trajectory 5 individuals, who had cardiac and renal problems, had the highest mortality with high event-free rates for kidney outcomes. Trajectory 6, which had a limited sample but demonstrated differences in body fat and alcohol, had the worst event-free rates for liver diseases. A detailed summary of these HyperTrajectories is provided in Figure 6, and individual examples are provided in Table S4.

### External Testing of HyperScore and HyperTrajectory

#### External Testing Data Set Characteristics

The ARIC data set that we used for external out-of-domain testing comprised 5507 participants who completed their fifth ARIC visit, which included brain MR examination (Table S5). As the aim of the analysis was to study time-to-event and survival, we also selected a subset that included all participants with a comparable age range to the UK Biobank (ie, 65 to 70 years of age). Within this subset, 568 (60.4%) were women. The use of ARIC also allowed us to test within a cohort with increased ethnic diversity (Figure S11). Of the selected group, 223 were classified as Black or African American (23.7%). Although ranges of HyperScore were similar across ethnic groups, mean HyperScore was significantly higher in the group of Black or African American individuals compared with individuals classified as White (*P*=0.020), with a larger difference in HyperScores between ethnic groups among women (*P*<0.001).

#### Testing Clinical Relevance Reproducibility

**Figure 7.**
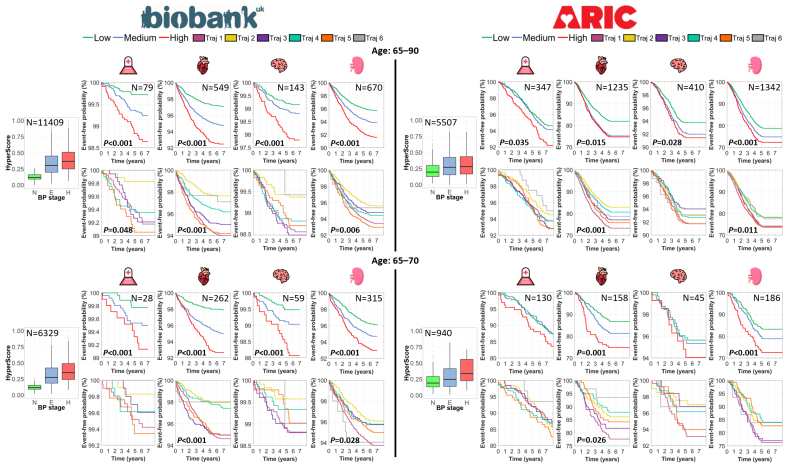
**External testing of HyperScore and HyperTrajectory on the ARIC study (Atherosclerosis Risk in Communities) data set (right) and comparison with the UK Biobank data set (left).** The predicted HyperScores relative to blood pressure (BP) groups along with survival (event-free) probability for death, cardiac, brain, and kidney events. Participants were also located on a HyperTrajectory, and survival curves were provided for each trajectory (Traj). ARIC data set was divided based on age range as 65–90 years (**top**; overall cohort) and 65–70 years (**bottom**; younger subset), and participants from the UK Biobank were selected according to the same ranges. The *P* value is reported only for significant curves and reflects overall statistical significance.

HyperScores generated for all individuals within the ARIC testing data set distributed across blood pressure levels in patterns similar to those observed in the UK Biobank (Figure 2D). Using the same internal validation approach as adopted for the UK Biobank, we found that a similar HyperScore threshold of 0.25 optimally differentiated those with no evidence of organ damage from those who would be defined based on the a priori criteria as having advanced end-organ disease. Additionally, comparison by JS distance showed similarity of HyperScore distributions across different levels of blood pressure within ARIC compared with the UK Biobank, especially for the H group, which reached as low as 0.10. Demographic variations and patterns of end-organ changes in the ARIC data set were also similar to those observed in the UK Biobank. Men tended to have higher HyperScores than women, and there were no significant differences in the curve characteristics of the main individual end-organ phenotypes between the 2 data sets. Time-to-event for mortality and other outcomes based on HyperScore ranges when classified as low, medium, and high (Figure 7, **top rows**) were similar between cohorts, with significant differences between each HyperScore category (Extended Results).

There was also a consistency of clinical findings within HyperTrajectories in ARIC compared with the UK Biobank (Figure 4C). The distribution of men and women across the HyperTrajectories was identical to the UK Biobank, with a predominance of women on trajectories 2, 4, and 6, whereas there was a predominance of men on trajectories 1, 3, and 5. Moreover, there were no significant differences in mean age, SBP, and DBP between ARIC trajectories. When comparing the phenotypical patterns of 2 key brain and heart measurements, namely white matter hyperintensities and LVEF, both had similar patterns across trajectories as observed across trajectories in UK Biobank. Similarly, using prospective follow-up data from ARIC, organ-specific events followed similar patterns of incidence across trajectories as observed in the UK Biobank (Figure 7, bottom rows). For example, ARIC participants in trajectory 1 had the worst survival patterns for cardiac events, and trajectory 5 had the highest mortality rates in both data sets, with particularly low survival rates for renal diseases (Tables S6 and S7).

## Discussion

For the first time, we have modeled a holistic representation of multiorgan phenotypic changes associated with hypertension by applying a novel contrastive approach to a large cross-sectional multimodal imaging data set. We found 6 predominant patterns of hypertensive end-organ disease with distinct demographic and outcome profiles. Furthermore, the severity and likely pattern of end-organ disease can be estimated for any new individuals by placing their data set within this landscape. These findings raise the possibility of personalized treatment plans for primary prevention in those with hypertension, which goes beyond blood pressure level, and takes account of current end-organ disease state.

A key feature of our semisupervised approach is its ability to discover multiple predominant patterns of phenotypic change in hypertension or HyperTrajectories. Unlike previous studies that focused on single-organ phenotyping (eg, cardiac,^35^ brain,^36^ or vasculature),^37^ our model captures hypertension as a systemic condition. For example, 2 HyperTrajectories with similar cardiac changes may still differ significantly in brain pathology, which could be developed to offer better insight into stratified prediction and treatment. However, such a discovery approach is inherently challenging because of a lack of ground truth. To address this, we evaluated model stability within the data set through multiple internal and external validation steps, our rationale being that if the models and trajectories identified are “true” representations of a pathological phenomenon of end-organ change, then the model should remain consistent in any data subset or external data set examined. As a sense check, we confirmed that known hypertension-related parameters, such as left ventricular hypertrophy, white matter lesions, and grey matter volumes, matched expected patterns across the HyperScore range.^35,38–40^ We further demonstrated stability across the HyperTrajectories, achieving sensitivity levels of up to 75% despite the complexity of a 6-class prediction task. This performance reflects the ability of the model to accurately reconstruct these patterns, even in the presence of strong similarities between individuals, particularly those with lower levels of organ damage.

The pseudotemporal interpretation of a cross-sectional data set with a gradual increase in severity of end-organ damage within the HyperTrajectory is fundamentally different from previous clustering approaches aimed at differentiating hypertensive disease states. The latter assumes a similar degree of end-organ change in all individuals.^41^ The 6 distinct HyperTrajectories emerged from a systematic, data-driven process that took into consideration the functional and structural changes of all major organs simultaneously. The discovery extends the current understanding of how hypertension can affect people in different ways and result in different outcomes. Although all event types occurred to some extent across trajectories, there were proportionally more events associated with the organs that were more affected in each trajectory (eg, cardiac events were more prevalent in trajectory 1, in which cardiac changes occurred earlier in the trajectory). Furthermore, although cardiac changes were also evident in HyperTrajectory 5, both 1 and 5 also displayed renal changes. Whereas HyperTrajectory 1 showed higher remodeling in cardiac function, HyperTrajectory 5 exhibited severe cardiac and renal pathology, which was linked to higher cardiorenal outcomes. Distinct underlying pathologies are likely to drive these 2 disease patterns, and this variation may have been difficult to identify if studying cardiac or renal changes in isolation.

There is now an opportunity to explore the distinct drivers and potential treatments for each HyperTrajectory, including individual genetic, lifestyle, or environmental factors. For example, current treatment guidelines often do not take variation by sex into account,^42^ whereas we found that women comprised the majority of individuals in HyperTrajectories 2, 4, and 6, whereas HyperTrajectories 1, 3, and 5 were predominantly composed of men. Consistent with existing literature, male-dominant trajectories had worse clinical outcomes including cardiac, vasculature, and pulmonary events compared with women.^43^ However, female-dominant trajectories had a sharper increase in white matter lesions with disease progression.^44^ Previous research has shown that incorporating imaging or organ changes into clinical decision-making with patients can significantly improve patient engagement.^45,46^ No such tool exists in hypertension management, and further work will be of value to understand whether use of end-organ severity or disease type can impact patient and clinician engagement, with management leading to improvements in outcome. Several new pharmacological strategies have emerged for hypertension management in recent years, including angiotensin receptor-neprilysin inhibitors (ARNIs), sodium-glucose cotransporter-2 inhibitors (SGLT2i), glucagon-like peptide-1 receptor agonists (GLP-1 RAs), and aldosterone synthase inhibitors.^47^ These agents demonstrate additional specific organ-protective effects that extend beyond blood pressure reduction, including attenuation of cardiac remodeling, preservation of renal function, and reduction of vascular and metabolic injury.^48^ Preliminary analysis of our available data has uncovered signals for different severities of organ disease, between trajectories, associated with certain prescribed medication (unpublished data). However, careful evaluation of treatment responses (eg, longitudinal assessment of changes in cardiac structure and function, renal biomarkers and glomerular filtration rate, and vascular and metabolic parameters, including through use of data available within randomized trials) will be necessary to determine whether these agents lead to meaningful modification or delayed progression of end-organ damage. Patient and treatment selection based on machine learning models of organ disease state may offer this information with targeted approaches for timely initiation and optimization of therapies before irreversible end-stage organ damage occurs.

As expected, the multiorgan summary provided by HyperScore outperformed carotid IMT, a single imaging measure,^49^ in prediction of future events, including in multivariable Cox modeling. HyperScore also provided prognostic information beyond blood pressure or traditional risk scores, particularly in those with elevated blood pressure. Traditional cardiovascular risk scores,^50^ which rely on demographic, laboratory, and blood pressure,^51^ often underperform in individuals with existing risk factors, younger individuals in whom the time horizon for events is less meaningful,^52^ and for capturing the full spectrum of hypertension-related outcomes. Although HyperScore was not trained to predict risk of future events and should therefore be disadvantaged compared with traditional scores optimized for risk prediction, it is striking that the performance of HyperScore was comparable to these scores. Indeed, HyperScore outperformed the scores in some tests while additionally providing personalized, organ-specific, and outcome-related assessments of disease severity. Risk scores may be disadvantaged over short time frames when compared with imaging tests, which can directly assess disease state. However, when we limited the time horizon to events occurring ≥4 years after assessment, HyperScore maintained its performance, consistent with the concept that the model is characterizing the full life course of hypertensive organ disease progression.

External testing in the ARIC participants also demonstrated stability of disease characterization and prediction of clinical outcomes in a geographically distinct and ethnically diverse cohort.^53^ Placement of individuals from the ARIC data set onto a HyperTrajectory also demonstrates a real-life scenario for applying the model to new patient data, especially given that ARIC individuals are typically older, at higher risk for cardiovascular diseases, and have had more events than represented in the UK Biobank.^33^ Differences in imaging modalities and settings used to acquire the measures in ARIC also did not appear to impact the stability of the model. For instance, echocardiography was used for cardiac measures in ARIC instead of cardiac MR, and ARIC used a 5-mm brain MRI slice thickness compared with 1 mm in the UK Biobank. Despite these technical variations, the proposed model appeared to remain resilient. Moreover, the diversity in the ARIC cohort enabled us to perform preliminary intersectional analyses by sex and ethnicity. In line with existing literature reporting higher rates of cardiovascular events and greater hypertensive target organ damage among individuals who classify themselves as Black, especially among women,^54^ we found that they also had significantly higher mean HyperScores.

There are several strengths to the contrastive learning methodology applied within this work. Our approach provides a multiorgan phenotyping framework, which, although it is not yet a fully validated risk stratification or progression model, allows for a more nuanced understanding of complex interactions across multiple organs. The contrastive semisupervised methodology enables isolation of variance within phenotypes uniquely related to hypertension, rather than general variance in the measures.^55,56^ In addition, to maximize the contrast between the group with normal organ phenotypes and those with hypertensive end-organ disease, we used an unsupervised isolation forest algorithm to remove patients with normal blood pressures but who may have “abnormal” healthy phenotypes, such as athletic remodeling or inherited phenotypic variation. Previous studies have primarily relied on blood pressure and time or age as sole criterions for determining disease state and progression.^57^ This can lead to limited phenotype characterization attributable to imprecise hypertensive disease burden categorization and tend to be only applicable to single organs.^35,58,59^ Moreover, compared with both simpler statistical models and more complex deep learning frameworks, the proposed cTI approach offers strong interpretability and reduced computational complexity while maintaining high performance.

### Limitations

This study has several limitations. First, the training cohort was drawn from the UK Biobank, which includes relatively “healthy” individuals of mainly White ethnicity. Although our modeled HyperScore and HyperTrajectory were applicable and showed similar associations with clinical outcomes in the ARIC data set, comprising nearly 20% Black or African American individuals, further studies are needed to assess ethnicity-driven differences in hypertension progression. Our initial ethnic analyses revealed a tendency for higher HyperScores in individuals classified as Black, particularly if they were women. Further research is needed to explore multiorgan disease development patterns across ethnicities. Second, mapping individuals within the end-organ disease landscape requires multimodality data, which are not widely available in clinical practice. We recently proposed a deep learning architecture to model complex data from simple measures that might be applicable to approximate HyperScore and HyperTrajectories from more accessible clinical measures, such as ECG data.^60^ Third, the model is cross-sectional, lacking a true temporal dimension, so further work will be of value to confirm temporal validity, identify potential reverse causation, and avoid overinterpretation of latent structure as disease progression. Although the map provides a pseudotemporal representation of disease severity based on organ damage, incorporating actual progression time (eg, age-related trajectories) could improve understanding of aging, duration of hypertension exposure, and organ function relationships. Future work could make use of repeat imaging and blood pressure measurements in longitudinal data sets to confirm the monotonicity of individual movement along trajectories. Fourth, trajectory 6 had a very limited sample size and poor stability in testing, therefore, although there did appear to be an increased frequency of liver-related outcomes, we would be cautious about interpretation of this trajectory until larger sample sizes are available. Finally, although the developed cPCA algorithm showed strong discrimination, unsupervised models continue to develop both within deep contrastive learning and as developments of contrastive PCA. These methods might offer improved dimensionality reduction, capturing nonlinear relationships and more granular phenotypes. However, given the interpretability and reduced computational complexity of the current approach, future development of simpler statistical approaches or deep learning frameworks should be evaluated, taking into account required tradeoffs between performance, model interpretability, and computational complexity.

### Conclusions

In conclusion, by applying novel computational approaches to large-scale imaging data sets, we have developed the first comprehensive and holistic model of end-organ changes related to hypertension. As such, this artificial intelligence (AI)–based cross-organ tool could lay the foundation for future reliable patient-specific hypertension assessment and personalized clinical management, leveraging AI insights into clinical practice.

## Article Information

### Disclosures

This work was conducted under UK Biobank application 58244 and ARIC accession No. HLB00020025a. P.L., W.L., A.L., and A.F. are named inventors on a filed patent describing the contrastive trajectory inference (cTI) approach for estimating cardiovascular disease progression (ID: WO2023041926). P.L. is a stockholder and founder of Ultromics, an AI and imaging company, and has received grants and/or provided consultancy for Ultromics, Pfizer, Astra Zeneca, Daiichi Sankyo, Novo Nordisk, Merck & Cie, Bracco, and Osler Diagnostics. No other authors declare competing interests. The UK Biobank data set is publicly available and can be accessed by application upon approval at: https://www.ukbiobank.ac.uk/enable-your-research/register. The ARIC data set can be acquired after approval from the National Heart, Lung, and Blood Institute at: https://aric.cscc.unc.edu/aric9/researchers/Obtain_Submit_Data. All algorithms and the developed model are integrated in the HyTwin toolbox, which can be accessed online at https://github.com/malkhodari/HyTwin_toolbox. The toolbox can be used to estimate the global organ damage score (HyperScore) and predict the likelihood of the organ-specific phenotype (HyperTrajectory) of any subset of individuals with any number of available multimodal imaging and nonimaging variables. The codes used to create the models and perform analysis were implemented on multiple programming platforms (Python, R, and MATLAB; details in Expanded Methods).

### Supplemental Material

Expanded Methods

*ICD-10* Codes

Extended Results

Figures S1–11

Tables S1–S10

Excel File (Data Sets Variables) S1

TRIPOD (Transparent Reporting of a Multivariable Prediction Model for Individual Prognosis or Diagnosis) Checklist

## Supplementary Material
