# Supplementary material for: Contrastive Machine Learning to Quantify Hypertensive Multiorgan Damage and Identify New Disease Phenotypes: A Multinational Multimodal Study
Source: Circulation. 2026 Jun 21;154(4):316–33. doi: 10.1161/CIRCULATIONAHA.125.077394 (PMC13399729; doi:10.1161/CIRCULATIONAHA.125.077394)
Supplement: Supplementary file 1 [file cir-154-316-s001.pdf]

Supplemental Material

EXPANDED METHODS

Dataset for model development

For model development, we used data from the Imaging Enhancement Study (2015-ongoing) of UK Biobank[13], an open-access resource that includes a large prospective cohort (age: 40-69 years old). The imaging substudy aims to collect multi-organ and multi-modality (brain, heart, and abdomen) scans of 100,000 participants. The study complies with the Declaration of Helsinki and was approved ethically by the Northwest Multi-Center Research Ethics Committee (MREC) as a Research Tissue Bank (RTB) approval (REC reference: 11/NW/0382) and received approval extension on 2021 (REC reference: 21/NW/0157). A written consent form was obtained from every participant prior to conducting the imaging study.

Our analysis included the currently available data of 46,102 participants. Each participant had 2,442 clinical measurements recorded using multiple tools including clinic resting blood pressure, brain MR, cardiac MR, abdominal MR, body composition, arterial stiffness, carotid ultrasound, spirometry, blood biochemistry, ECG, and baseline/demographic characteristics. Full details of the measurement protocols have been described previously[13]. Moreover, all these variables were imaging derived and did not include any raw imaging data.

Data Curation and Cohort Selection

Pre-processing and Data Preparation

All participants with missing blood pressure measurements or more than 5% of missing variables were removed (n = 18,038). In addition, participants who have suffered from prior heart attack, angina, or stroke (n = 965) were also removed. Variables with more than 50% missing values were removed from the dataset (n = 1,388). The final dataset included 27,099 participants with 1,054 multi-modality measurements. Any values above 5 standard deviations

(SD) above or below the mean, *i.e.*, outliers, were removed and replaced as missing. The median of the selected variable then imputed all remaining missing values.

Feature Selection to Reduce Redundancy

We performed feature selection to reduce redundancy and enhance variability across each modality. The selection was based on reducing the overall covariance between variables where we excluded one of the two variables with covariance above 95%. This has a major impact on modalities with many variables, *i.e.*, brain MR and body composition, which required additional selection through contrastive covariance analysis. A contrastive covariance matrix was computed as the subtraction between the normotensive and hypertensive covariance matrices so that variables greater than twice of the standard deviation were excluded. Accordingly, variables were reduced from 1,054 to 566 collecting most of the complex relationships at every modality (**Figure S1**); it contained 449 brain MR, 13 cardiac MR, 12 carotid ultrasound, 24 body composition, 58 blood biochemistry, 3 ECG, 5 spirometry, and 2 demographic characteristics. The list of all variables and modalities used in this study is provided in **Supplementary Material**.

Adjusting Covariates and Harmonization

We adjusted for covariates to reduce the effects of confounding factors, *i.e.*, age and sex, that were not controlled during the study. We used robust linear regression to residualize each feature on the covariates, and significant covariate effects were subtracted from all subjects to obtain adjusted features. Any missing covariate values were mean-imputed. In addition, we used the recent ComBat tool[14] to perform data harmonization across the three imaging centres where the imaging substudy of the UK Biobank was conducted. Harmonization was important to reduce the variance caused by non-biological sources in multi-centre studies.

Definition of Groups for Contrastive Analysis

The proposed study requires contrastive modelling using participants considered the 'healthiest', *i.e.*, having similar measurement values representing the healthy state, and comparing these to participants likely to be representative of advanced disease state. In this analysis, the 'healthy' state includes individuals who would not be expected to have evidence of hypertensive end-organ damage, and we refer to these as normotensive (N). We defined this "N" group as participants having systolic and diastolic blood pressure below 120/80 mmHg during their first imaging visit. In addition, they had never received any blood pressure medications and had never been diagnosed with hypertension.

In addition, as several of the "N" group despite having normal blood pressure levels may still have exhibit altered multi-organ clinical measurements due to other factors such as athletic performance or inherited abnormalities, we automatically identified any outlier participants in this group (the N+ group), using an isolation forest algorithm[15]. A participant was assigned as N+ if the outlier probability given by the isolation forest was more than the third quartile of all probabilities assigned to each participant. This reduced the "N" group by a further 344 individuals (**Figure 1b**). "N" and "N+" groups were significantly different in key organ measurements.

For the contrastive analysis, we then identified the group most likely to have evidence of end-organ disease and refer to them as the hypertensive end-organ group (H). These participants had either a systolic or diastolic blood pressure of more than 160 or 100 mmHg, respectively, in line with stage 2 hypertension as defined by the 2014 Eighth Joint National Committee (JNC 8) guidelines[16], 2017 American Heart Association/American College of Cardiology (ACC/AHA) guidelines[17], 2023 European Society of Hypertension (ESH) guidelines[18]. Both the normotensive and hypertensive groups were determined using blood pressure thresholds

that are common across all management guidelines. All remaining participants were assigned to an intermediate elevated group (E).

Model Development

Dimensionality Reduction and cPCA

To capture the most informative components in the data and reduce dataset dimensionality, we used a cPCA algorithm[19]. Contrary to the conventional PCA algorithm, cPCA performs weighted subtraction (**Figure S2**) between individual covariance matrices of the two extremities, *i.e.*, group N and group H, to form the contrastive covariance matrix (cov_c) as follows,

$$cov_c = cov_h - \alpha * cov_n$$

where cov_h and cov_n are the covariance matrices of the "H" and "N" groups, respectively, and α is the contrast parameter that quantifies the trade-off between having high "H" and low "N" variance, and thus, disease variance dominates.

In this study, a logarithmic range of 50 weighting factors from $\alpha = 10^{-2}$ to 10^{1} was applied to the weighted subtraction, and the optimal factor was determined iteratively based on an evaluation of the ability of the model to differentiate group "H" from group "N". This performance testing allowed us to identify settings that allowed the highest discrimination between the two groups, which had been predefined to represent the two most different end-organ disease states. On each iteration, regular PCA was performed and the optimal α was determined when there was a high clustering tendency between "N" and "H" groups (using spectral and *k*-means clustering). The eigenvectors produced from the optimal α were the final reduced contrastive principal components (cPCs) for the dataset. Here, the combination of

components that most effectively differentiates the two contrastive groups through spectral and *k*-means clustering will be automatically identified and used as the reduced dimension.

Accordingly, variable importance (C_i) to the modelling was calculated based on the acquired eigenvectors[20] as follows,

$$C_i = 100 \times \left(\frac{\omega_i^2}{\sum_{k=1}^{K} \omega_k^2} \right) \times \lambda$$

where ω_i^2 is the eigenvectors of the selected variable i, $\sum_{k=1}^{K} \omega_k^2$ is the sum of all eigenvectors at every component (k), K is the total number of cPCs, and λ is the eigenvalues.

The most important variables were determined using a cut-off threshold of the expected contribution value ($C_{expected}$) as follows,

$$C_{expected} = 100 \times \left(\frac{\sum_{k=1}^{K} \lambda_k}{N} \right)$$

where N in this equation represents the total number of variables.

Contrastive Trajectory Inference (cTI)

Upon obtaining the reduced dimensional cPCA space, we performed cTI[20] by first calculating the Euclidean distance matrix of the cPCA components among all participants, from groups "H", "E" and "N". Then, we automatically selected the healthiest participant, *i.e.*, the closest to all other "N" participants in the distance, as the root node. This individual represented the data point that was the closest to all other healthy individuals, which is considered to be the healthiest with less variability relative to the healthy state. The root node was then used to create a minimum spanning tree (MST)[21] un-directed graph with the aim of forming optimal connections that minimize the summed length of all paths from the root node. The distance of each participant from the root node was generated within a normalized (range: 0—1) relative

to the maximum value (the farthest distance from the root node) that should represent the extreme end-organ condition. This value thereby provides a quantitative measure of end-organ disease state, *i.e.*, the proximity of each participant from the pathology-free state relative to the most advanced disease state.

Clustering Graph Connections

As participants were not distributed homogenously through the cPCA space, we also investigated whether there were dominant 'clusters' of participants tracking through the space from the root node to various advanced disease states, *i.e.*, 'trajectories' of end-organ change within the space. To do this, we identified dominant contrasted pseudo-temporal connections in the MST. We used Laplacian transformation[22] to create an embedding Eigenspace with no dimensional meaning for the axes.

We then defined major pathways in the constructed graph by following three major steps. First, minor pathways with more than 95% overlap with any other 'trajectory' were combined. Then, the objective merging of highly overlapping 'trajectories' was performed iteratively, where on each iteration, the highest overlapping two 'trajectories' were determined and combined as one. This was performed until 25 'trajectories' remained. As some of these 'trajectories' contained a very small number of participants, further combinations were then performed of highly overlapping 'trajectories', until each major 'trajectory' included at least 10% of the participants; a threshold which we chose a priori to avoid bias in trajectory definition. After this step, six 'trajectories' were definable (**Figure S3**).

Generation of HyperScore and HyperTrajectory

Key Definitions

For ease of description, we refer to the distance of each participant from the root node, *i.e.*, the quantitative measure of end-organ disease state as their, *HyperScore.* Similarly, we also

describe the 'trajectory' that each participant was allocated to as their, *HyperTrajectory*. To simplify group comparison, we also allocated participants to three *HyperScore* stages based on the distribution of scores within the "H" group, being the group defined as likely to have end-organ damage. The first 'minimal end-organ disease' stage was thereby defined by a *HyperScore* between 0 and the first quartile (Q1) of participants within the "H" group. This threshold was also consistent with the threshold identified in a Youden index analysis as the value that most effectively discriminated the "N" from the "H" groups. The second stage, 'medium end-organ disease' stage, was the range of scores between Q1 and the third quartile (Q3) of the "H" group. Lastly, the third stage, 'high end-organ disease' stage, is the range of scores between Q3 and the maximum of all scores. We evaluate the contrastive separation performance, *i.e.*, model's ability to discriminate between the N and H groups, using the area under the receiver operating characteristic curve (AUC). Here, the analysis was performed using the assigned scores for the N and H groups.

Predictions for 'Unseen' Individuals

In addition, we developed methodology to assign a *HyperScore,* and identify the most likely *HyperTrajectory*, for participants who were not included in the development of the baseline model. To estimate a *HyperScore* for hidden participants, *e.g.*, those in any subsequent 'testing dataset' or new patients in any clinical application, we used a *k*-nearest neighbour (*k*-NN) approach[23]. Here, we first transformed hidden data using the pre-existing cPCA space to form their principal components. Then, a *k*-NN of 25 neighbours was used to find the most similar participants in the original dataset to the hidden data. A *HyperScore* was assigned accordingly as the mean of the closest 25 participant scores. The selection of *k* to be 25 was based on quantitative analysis to determine the optimal number of close participants to the testing individual while ensuring less variability between their cPCs. Moreover, this value was selected as a compromise, where lower values of *k* are sensitive to local variations, whereas higher

values overly smooth the probabilities and can be influenced by distant neighbours[24]. Similarly, by using a k-NN approach we were able to determine the likelihood for a participant to be on any *HyperTrajectory* based on the allocation of each nearest neighbour to different trajectories. As it was possible that not all 25 nearest neighbours were allocated to the same *HyperTrajectory,* particularly if participants were positioned nearer to the root node, where there was less distance between trajectories, participants could be provided with a likelihood, or probability, for allocation to each trajectory.

Internal Validation

Stability of HyperScores Generation

To understand the stability of the modelling approach as a quantitative measure of end-organ changes, we performed internal validation by building models based on smaller subsets of the full dataset. We then used this model to predict *HyperScores* for participants in the held-out data. The re-generated *HyperScore* was compared with the score generated originally when modelling with the full dataset, and size of differences between scores evaluated using root mean square error (RMSE), with an expectation that there would be <10% variation between scores. We did this in two ways. Firstly, using a stratified (sex and blood pressure) 10-fold cross-validation scheme. A random 10% of the input data was removed at each iteration, and the modelling was performed to generate a new set of *HyperScore* for the remaining participants. Secondly, we performed this testing step by building two separate models based on half the available data. We did this by splitting the data randomly into two datasets representing 50% of the participating cohort and also by splitting the dataset into two separate models based on the time of the imaging visit, namely from 2015 to 2018 and from 2018 to 2021. Normalization and data imputation were performed on each subset separately prior to

modelling. This ensured that there is no data leakage during the development and prediction of held-out sets of the cross-validation.

As an additional exploration of the stability of the modelling in the absence of ground-truth, we also followed a confidence-based performance estimation (CBPE) approach[25]. Here, performance was determined by means of the overall probability towards a certain condition, *i.e.*, presence of hypertensive end-organ changes. This test is based on where an individual was positioned relative to the optimal threshold previously identified for differentiation of *HyperScore* range between 'no end-organ disease', or group "N", and 'presence of end-organ disease', or group "H". It allows for determining an optimal threshold to group participants based on end-organ damage, independently of blood pressure grouping. The generated *HyperScores* were used as the predicted probability. A confusion matrix was created to calculate performance metrics based on the sum of calibrated probabilities.

We further evaluated stability by studying 'data drift' when continuously adding new data to the trained model. To evaluate this, we first held out 10% of the data as a test set, trained the model on 50% of the remaining data, and kept adding 10% iteratively (5 iterations). At each iteration, the RMSE was calculated between the first modelled *HyperScores* of the test set and the new scores generated after the addition of new data. The same approach was evaluated for the CBPE metrics (optimal threshold and AUC), where we started with 20% of the data, then continuously added 20%, and observed the changes.

Additionally, to take account of the full distribution of predictions for each individual we also evaluated performance using Jensen-Shannon divergence[26] which compares two probability distributions to measure their similarity. The divergence (JS) is calculated between two probability distributions (P) and (Q) as follows,

$$JS(P||Q) = \frac{KL(P||M) + KL(Q||M)}{2}$$

Where KL is Kullback-Leibler divergence[27] and $M = (P + Q)/2$ is the matrix distribution

JS is bounded by 1 for two probability distributions, given that the base 2 logarithm is used. In general, the lower the JS value, *i.e.*, close to zero, indicates greater similarity between the two distributions, whereas the higher JS value, *i.e.*, close to 0.7, indicates dissimilarity between the two distributions. Here, we compared between the distribution of probabilities for the original *HyperTrajectory* and the per-fold trajectories for the three *HyperScore* stages defined earlier.

Stability of HyperTrajectory Allocation

To understand the stability of the modelling as an approach to identify clusters of individuals with similar patterns of end-organ changes, we performed internal validation with stratified 10-fold cross-validation. As the trajectories rely on identification of paths between the root node and most extreme end-organ disease state, we ensured the training set always included the 5% of individuals closest to the root node, *i.e.*, healthiest participant in the dataset, and the 5% of participants representing the most severe form of end-organ changes within the full dataset. We then used the MST graph created with each fold of data and regenerated six major pathways within that data. Accordingly, each individual within the held-out data was then attributed a probability for location on each trajectory, their *HyperTrajectory* probability. This allocation was compared to their original attribution within the full model.

As each individual is attributed probabilities across the six trajectories, we firstly evaluated similarity simply based on prediction of the top-2 most likely trajectories, *i.e.*, a prediction is considered correct if it was predicted among the highest two predicted trajectories. A confusion matrix was constructed, and the performance metrics were calculated relative to the ground truth, *i.e.*, the original model.

Sensitivity Analysis of HyperScore and HyperTrajectory

To evaluate the robustness of the *HyperScore* and *HyperTrajectory*, we conducted additional sensitivity analyses by excluding selected variables prior to model development and examining the resulting changes in performance. Robustness was assessed in terms of both individual-level *HyperScore* estimation and prediction of individual *HyperTrajectory* allocation probabilities. Specifically, we excluded three key variables: left ventricular ejection fraction (LVEF), white matter hyperintensities (WMH), and creatinine, as well as two entire modality blocks, cardiac MR and brain MR, together with all their associated variables.

For *HyperScore*, robustness was quantified by calculating the RMSE between scores derived from the full model and those obtained under each exclusion scenario. For *HyperTrajectory*, robustness was assessed using the JS distance between trajectory allocation probabilities estimated from the full model and those derived from each reduced model.

Additionally, when the full cardiac MR and brain MR variable blocks were excluded, we performed event-free probability analyses for cardiac and brain adverse outcomes and compared these results with the corresponding estimates from the original model.

Clinical Relevance of *HyperScore*

To test whether the *HyperScore* had biological and clinical relevance, we first visually assessed how individual clinical phenotypes known to vary with exposure to hypertension varied across the *HyperScore* range to sense check direction and shape of association. We then hypothesised that individuals with more advanced end-organ changes should be closer to having a hypertensive-related event than individuals who may have similar blood pressure but have less evidence of end-organ disease. To test this hypothesis, we used prospective follow-up data collected on individuals who had participated in the UK Biobank Imaging Enhancement and studied time to event within each *HyperScore* range, *i.e.*, low, medium and high using Kaplan-

Meier[27] and log-rank (Mantel-Cox) testing with a significance threshold of 0.05. The diagnosis was derived from the International Classification of Disease Version 10 (ICD-10) reports derived from Hospital Episode Statistics (HES) data (**Supplemental Material**) and analysed as an event-free probability. All outcomes were utilised for discovery and analysis and were not involved in model development. As these outcomes were not algorithmically defined, no event adjudication was required.

Additionally, to evaluate the independence from blood pressure level, we studied the time-to-event with the study group stratified by blood pressure level in two ways. Firstly, in four groups, with participants grouped as normotensive and with a low HyperScore (NL), normotensive and medium- or high-HyperScore (NMH), elevated or hypertensive and low-*HyperScore* (EHL), and elevated or hypertensive and a medium- or high-*HyperScore* (EHMH). Secondly, in nine groups, participants were grouped as normotensive and low- *HyperScore* (NL), normotensive and medium- *HyperScore* (NM), normotensive and high- *HyperScore* (NH), elevated and low-*HyperScore* (EL), elevated and medium- *HyperScore* (EM), elevated and high- *HyperScore* (EH), hypertensive and low- *HyperScore* (HL), hypertensive and medium- *HyperScore* (HM), and hypertensive and high- *HyperScore* (HH).

As a final evaluation of clinical relevance, we compared how effectively *HyperScore* predicts time-to-event, compared to widely available 'gold standard' risk models, optimised to predict future events, *e.g.*, QRISK3, ASCVD, Framingham, MESA, as well as imaging measures proposed as predictive risk markers, *i.e.*, carotid IMT. We further fitted multivariable Cox models incorporating the score, age, and sex and report hazard ratio (HR) per SD (with 95% CI and p-value), C-statistic (score, age+sex, and score+age+sex), and ΔC-statistic. Moreover, we assessed model calibration using a 10-fold cross-validation scheme and calculated calibration correlation, intercept, slope, net reclassification improvement (NRI), and integrated discrimination improvement (IDI)[30]. We compared *HyperScore* versus blood pressure (systolic

and diastolic) and *HyperScore* versus established risk scores. We included the quantified results for circulatory diseases and deaths, and we added also separated brain diseases and circulatory deaths for additional outcome analysis. As an additional analysis of the ability of *HyperScore* to predict long-term outcomes, we estimated time-to-event probabilities for circulatory diseases and death by applying a threshold of 4 years after the imaging visit and including only events occurring within this time frame for all scores.

Clinical Relevance of *HyperTrajectory*

To study whether the *HyperTrajectory* provided additional biological and clinical relevance beyond that obtained from the *HyperScore* alone, we examined demographic information, disease outcomes, and imaging phenotypes across the six *HyperTrajectories*. We summarised the trajectories by identifying distinct patterns evident across them. Here, we allocated to each *HyperTrajectory* the predominant characteristic evident, *i.e.*, the major end-organ-related attribute that had the most marked differences at the highest 5% *HyperScores* within that *HyperTrajectory,* which represents the advanced disease state. The criteria used to determine the predominant characteristic were as follows,

1) ***Diseases distribution:*** we used ICD-10 codes to determine the proportions of diseases such as cardiac, brain, vasculature, pulmonary, liver, kidney, and metabolic diseases, alongside circulatory-related mortality, across the *HyperTrajectories*.
2) ***Time-to-event:*** we looked for event-free probabilities related to the aforementioned diseases affecting multiple organs within each *HyperTrajectory*.
3) ***Phenotypical changes:*** we identified significantly different imaging variables using a linear mixed effects model adjusted for age and sex. These phenotypes were then examined to validate outcome-related findings and to determine potential associations with the targeted organ-related changes.

External Testing

External Testing Dataset

We sought to formally test, on an independent external dataset, the reproducibility of the clinical relevance of *HyperScore* and *HyperTrajectory* observed in the training and validation steps. For this external testing we used data collected within the Atherosclerosis Risk in Communities (ARIC) study[31]. ARIC is a population-based surveillance study for myocardial infarction (MI) incidence and coronary heart disease (CHD) mortality in the United States. The main goal of the study had been to understand the risk factors for subclinical atherosclerosis. The study launched in 1985 in four geographically diverse regions, i.e., Forsyth County – North Carolina; Jackson – Mississippi; suburban Minneapolis – Minnesota; and Washington County – Maryland. All four centres approved ARIC protocols through their institutional review boards, and participants provided written consent before the enrolment. Recently, ARIC data bec ame part of the data-sharing efforts in the National Heart, Lung, and Blood Institute (NHLBI) TransOmics for Precision Medicine (TOPMed) program[33].

Nearly 16,000 adults (4,000 from each region) aged between 45 and 64 years old were enrolled prospectively in the study comprising white and black-African Americans early in 1987 and followed up for more than 35 years through multiple visits, which are identified numerically, in chronological order, from visit 1 to 12. At the early visits, *i.e.*, visits 1 to 3, participants provided demographic and anthropometric data, medical history and lifestyle, clinical laboratory measurements, genetic data, cognitive and mental health details, and hospitalisation and mortality surveillance. From visit 3 onwards, participants performed modality-based examinations such as 12-lead ECG, brain MR, retinal imaging, ultrasound and carotid IMT measurements, echocardiography, and spirometry.

For our external testing, we focused on participants in visit 5, *i.e.*, between 2011 and 2013, because this visit involved brain MR examinations. We initially performed a comparative analysis of end-organ metrics in ARIC compared to UK Biobank to ensure feasibility of model testing. The majority of available measures in ARIC had been collected using comparable methods to those used in UK Biobank. However, there were notable differences. Echocardiography was used within ARIC, as opposed to cardiac MR in UK Biobank. However, as the set of standard cardiac measures available in UK Biobank can also be generated from echocardiography, *e.g.*, LVS and LVED, we substituted the echocardiography variables for cardiac MR measures. In addition, there was a significant difference in brain MR slice thickness between ARIC and UK Biobank. As the identified variation in cardiac and brain indices between studies can lead to recognised differences in normal ranges for these measures, we allowed for this variation in interpretation, and using normalised ranges when comparing between datasets where appropriate. Following this analysis, our identified testing dataset included 123 variables that matched variables used in the original UK Biobank model. These included 75 brain MR, 7 cardiac imaging variables, 5 body composition, 26 blood biochemistry, 3 ECG, and 5 spirometry, in addition to 2 demographic characteristics, *i.e.*, age and sex. The list of all variables and modalities used in this study from ARIC is provided in **Supplementary Material**. It is worth noting that no calibration was required for the ARIC dataset, as the modelling approach was not based on linear regression or conventional predictive modelling with estimated probability outputs. Instead, the framework relies on cPCA/MST–based modelling, which does not require recalibration when applied to new datasets.

External Testing of Clinical Relevance

To externally test the clinical relevance, we first generated a *HyperScore* and probability of allocation to *HyperTrajectory* for all participants in the ARIC testing dataset, using the method

described earlier for attribution of these features to unseen data, using normalised variables to account for variation in normal ranges between studies. ARIC variables were normalized based on the UK Biobank corresponding variables, *i.e.*, using their mean and standard deviation values. *HyperScores* and *HyperTrajectories* were based on available variables per-participant, for which the corresponding cPC coefficients were selected from the overall cPCA space to apply the transformation. We then performed the exact same protocol for evaluation of clinical relevance of *HyperScore* and *HyperTrajectory* as used in the internal clinical validation. This included visual assessment of the change in individual phenotypes with increasing *HyperScore* and study of time-to-event according to *HyperScore* range. We then looked at *HyperTrajectory* allocation and studied variation in individual phenotypes and time-to-event by *HyperTrajectory*. All findings were compared to those observed in the internal validation to evaluate the consistency of clinical findings in the external dataset.

Statistical Methods and Regression Analysis

If not otherwise indicated, all statistical tests in this study were carried out using the analysis of variance (ANOVA) with a significance level of 0.05. To evaluate phenotypic patterns, we performed regression analysis using locally estimated scatterplot smoothing (LOESS) fitting and compared it with linear fitting. Error percentage was provided as the overall ratio of RMSE between data points and the fitted line. Linearity analysis was performed by evaluating the coefficient of determination (r^2) provided by the fitted line to the data points. Moreover, the *p*-value shows the significance of this fitting relation. We performed statistical analysis over the fitted line coefficients to compare models during the validation of phenotypic patterns. The statistical analysis of variables predicting trajectories was performed using a linear mixed effects model adjusted for age and sex.

Programming Packages Used for the Analysis

Data preparation and preprocessing were conducted in RStudio (version 2022.7.2.576). The cTI algorithm was implemented in MATLAB R2021a, using the Machine Learning Toolbox for functions related to spectral clustering, k-means clustering, and MST graph generation. Trajectory characterization was performed using Python (version 3.13.5).

ICD10 codes used in survival analysis

Cardiac—

- **Chapter IX Diseases of the circulatory system**
    - **I10-I15 Hypertensive diseases**
        - ♣ I11 Hypertensive heart disease
    - **I20-I25 Ischemic heart diseases-**
        - ♣ I20 Angina pectoris
        - ♣ I21 Acute myocardial infarction
        - ♣ I22 Subsequent myocardial infarction
        - ♣ I23 Certain current complications following acute myocardial infarction
        - ♣ I24 Other acute ischemic heart diseases
        - ♣ I25 Chronic ischemic heart disease
    - **I30-I52 Other forms of heart disease**
        - ♣ I46 Cardiac arrest
        - ♣ I50 Heart failure

Brain—

- **Chapter IX Diseases of the circulatory system**
    - **I60-I69 Cerebrovascular diseases**
        - ♣ I60 Subarachnoid hemorrhage
        - ♣ I61 Intracerebral hemorrhage
        - ♣ I62 Other nontraumatic intracranial hemorrhage
        - ♣ I63 Cerebral infarction
        - ♣ I64 Stroke, not specified as hemorrhage or infarction

        - ♣ I66 Occlusion and stenosis of cerebral arteries, not resulting in cerebral infarction
        - ♣ I67 Other cerebrovascular diseases
        - ♣ I68 Cerebrovascular disorders in diseases classified elsewhere
        - ♣ I69 Sequelae of cerebrovascular disease

Vasculature—

- **Chapter IX Diseases of the circulatory system**
    - **I70-I79 Diseases of arteries, arterioles and capillaries**
        - ♣ I70 Atherosclerosis
        - ♣ I71 Aortic aneurysm and dissection

Liver—

- **Chapter XI Diseases of the digestive system**
    - **K70-K77 Diseases of liver**
        - ♣ K70 Alcoholic liver disease
        - ♣ K71 Toxic liver disease
        - ♣ K72 Hepatic failure, not elsewhere classified
        - ♣ K73 Chronic hepatitis, not elsewhere classified
        - ♣ K74 Fibrosis and cirrhosis of liver
        - ♣ K75 Other inflammatory liver diseases
        - ♣ K76 Other diseases of liver
        - ♣ K77 Liver disorders in diseases classified elsewhere
    - **K80-K87 Disorders of gallbladder, biliary tract and pancreas**
        - ♣ K80 Cholelithiasis

        - ♣ K81 Cholecystitis
        - ♣ K82 Other diseases of gallbladder
        - ♣ K83 Other diseases of biliary tract
        - ♣ K85 Acute pancreatitis
        - ♣ K86 Other diseases of pancreas
        - ♣ K87 Disorders of gallbladder, biliary tract and pancreas in diseases classified elsewhere

Kidney—

- **Chapter IX Diseases of the circulatory system**
    - **I10-I15 Hypertensive diseases**
        - ♣ I12 Hypertensive renal disease
- **Chapter XIV Diseases of the genitourinary system**
    - **N00-N08 Glomerular diseases**
    - **N10-N16 Renal tubulo-interstitial diseases**
    - **N17-N19 Renal failure**
    - **N20-N23 Urolithiasis**
    - **N25-N29 Other disorders of kidney and ureter**
    - **N30-N39 Other diseases of urinary system**

Metabolic—

- **Chapter IV Endocrine, nutritional and metabolic diseases**
    - **E00-E07 Disorders of thyroid gland**
    - **E10-E14 Diabetes mellitus**
    - **E15-E16 Other disorders of glucose regulation and pancreatic internal secretion**

    - **E20-E35 Disorders of other endocrine glands**
    - **E40-E46 Malnutrition**
    - **E50-E64 Other nutritional deficiencies**
    - **E65-E68 Obesity and other hyperalimentation**
    - **E70-E90 Metabolic disorders**

Pulmonary—

- **Chapter IX Diseases of the circulatory system**
    - **I26-I28 Pulmonary heart disease and diseases of pulmonary circulation**
        - I26 Pulmonary embolism
        - I27 Other pulmonary heart diseases
        - I28 Other diseases of pulmonary vessels

EXTENDED RESULTS

Internal Validation

Stability of HyperScore Estimation

When randomly splitting the data for training of models A and B, MRMSE values (**Figure 2b**) did not significantly differ between the modelled and predicted *HyperScores* for individuals in both approaches (A: 0.13±0.01, B: 0.14±0.01). Moreover, when splitting the data for training of two models based on time of the imaging visit, both models had low MRMSE values of 0.16±0.08 and 0.17±0.07, respectively, whether compared to *HyperScores* generated in the combined model or when using set 1 to build the model and set 2 as the predicting dataset. In this case, the MRMSE value was 0.14±0.03 and the opposite setting resulted in a MRMSE of 0.16±0.05.

Analysis of CBPE showed an optimal *HyperScore* threshold of T = 0.28 to differentiate groups (**Figure S5a**), having the highest sensitivity and specificity levels (66.2% each) with true positive and true negative ratios of 0.196 and 0.466, respectively, and an overall AUC of 0.72. The drifting effect (**Figure S5b**) was minimal when considering the impact on the optimal threshold for differentiation of the N and H groups (±0.007) and AUC (±0.023) values in the CBPE during the continuous addition of data to the model. Finally, evaluation of MRMSE demonstrated the measure remained low at 0.11±0.11, when iteratively adding data to the training dataset and comparing to the test dataset in the data drifting analysis, consistent with a stable model.

The evaluation of performance using the JS distance[26] metric (**Figure S5c**) showed the high-risk group always had the lowest average distance across folds compared to the full model, with 0.27±0.04 and 0.24±0.05 for the stability and prediction tests, respectively. On the other

hand, the highest average distance values were found for the low-*HyperScore* group for stability and prediction with 0.38±0.04 and 0.33±0.04, respectively.

Sensitivity Analysis of HyperScore and HyperTrajectory

When excluding LVEF, WMH, and creatinine simultaneously during model development, the difference in *HyperScore* between the full baseline model and the reduced model was minimal (RMSE = 0.093). When these variables were excluded individually, the RMSE values were 0.061, 0.088, and 0.087 for the LVEF-, WMH-, and creatinine-excluded models, respectively (**Table S8**). For *HyperTrajectory*, exclusion of all three variables resulted in a JS distance of 0.273, while exclusion of LVEF, WMH, and creatinine individually yielded JS distances of 0.269, 0.308, and 0.283, respectively. With regard to modality exclusion (**Table S8**), *HyperScore* exhibited an RMSE of 0.097 when cardiac MR variables were excluded and 0.144 when brain MR variables were excluded. For *HyperTrajectory*, excluding cardiac MR and brain MR resulted in JS distance values of 0.264 and 0.340, respectively.

Clinical Relevance of *HyperScore*

Comparison with Conventional Prediction-based Tools

Comparison of our machine learning- and imaging-based metric of current end-organ disease state, as a predictor of time-to-event, compared to clinical scoring systems confirmed *HyperScore* had comparable predictive ability for future events (**Figure S7**), particularly circulatory deaths. In addition, *HyperScore* effectively predicted time-to-event for a range of other individual clinical outcomes relevant to hypertension, whereas performance of established risk scores and carotid IMT was less consistent across conditions.

Clinical Relevance of *HyperTrajectory*

Sensitivity Analysis of time-to-event

Excluding the entire cardiac MR or brain MR blocks of variables did not substantially alter the trajectories' ability to predict outcomes (**Figure S10**). Excluding the cardiac MR block reduced Trajectory 1's (cardiac-dominant) ability to discriminate cardiac event rates and pushed the estimation of brain outcomes rate for Trajectory 4 (brain-predominant). On the other hand, excluding the brain MR block did not alter cardiac outcomes estimations. It primarily affected Trajectory 4's (brain-predominant) ability to estimate brain outcomes rates.

External Testing of *HyperScore* and *HyperTrajectory*

External Testing Dataset Characteristics

The ARIC dataset comprised 5,507 participants who completed their 5th visit. The selected cohort had median age of 75 years old (72—80) with a mean arterial pressure (MAP) of 87 mmHg (80—95). Moreover, the dataset included 3,176 female participants (57.67%). We selected a similar subset in terms of age range from the UK Biobank cohort, i.e., 65 to 95 years old, which are the minimum and maximum in the ARIC cohort. Accordingly, among the selected 11,409 individuals, their median age was close to ARIC at 70 years old (68—73), MAP significantly higher at 101 mmHg (93—109), and female proportions slightly lower of 5,590 (49.00%).

As the aim of the analysis was to examine time-to-event and survival, we selected a subset from ARIC that included all participants within an age range comparable to the UK Biobank, i.e., 65 to 70 years. This range represented the lowest ages in ARIC and the third quartile of ages in the overall UK Biobank cohort. The ARIC subset included 940 individuals, of whom 568 (60.4%) were female. In contrast, the same age range in the UK Biobank comprised 6,329 individuals, including 3,248 females (51.32%). Regarding MAP, the UK Biobank consistently showed higher blood pressure than ARIC, with a median of 100 mmHg (92—108) compared to 87 mmHg (80—95), respectively.

The use of ARIC also allowed us to test within a cohort with an increased diversity. In the overall cohort, 19.38% were of Black-African American ethnicity (1,067 individuals), whereas the younger cohort had 23.72% (223 individuals). When compared to their UK Biobank counterparts, 0.28% was included as black ethnicity in the two selected cohorts. Details of the ARIC cohort from visit 5 and the subsets used for external testing are provided in **Table S4**.

HyperScore Prediction Similarities

The distribution of *HyperScores* in ARIC across the three groups was consistent with that observed in the UK Biobank counterpart (**Figure 2d**). Using JS distance to quantify similarity, the overall cohort showed the greatest alignment at the intermediate "E" group (JS distance = 0.14), followed by the target "H" group at 0.28. The background "N" group exhibited the largest divergence at 0.48. In the younger subset, the closest match was observed for the target "H" group at 0.17, followed closely by the intermediate "E" group at 0.18, while the background "N" group showed the greatest distance with 0.48.

We further assessed score distributions by sex and observed similar patterns across male and female groups of individuals, although men consistently exhibited significantly higher HyperScores compared to women in the UK Biobank cohort. This sex-related difference was similar but less pronounced within the ARIC cohort. To evaluate clinical correlation, we examined the progression of organ damage (after z-score normalisation) in relation to predicted HyperScores for BMI, LVS, and white matter hyperintensities. These variables were selected because they are important for the modelling and are well established clinically in the context of hypertension. In the overall cohorts, slope comparisons between datasets were nonsignificant (BMI: p = 0.135; LVS: p = 0.242; WMH: p = 0.248). Similarly, in the younger subset, slopes did not differ significantly (BMI: $p = 0.476$; LVS: $p = 0.053$; WMH: $p = 0.297$).

Testing Clinical Relevance Reproducibility

We evaluated time-to-event outcomes for mortality and major adverse events, including cardiac, brain, and kidney diseases, stratified by *HyperScore* categories, i.e., low, medium, and high. The event-free probability curves demonstrated consistent patterns across cohorts, with significant differences observed between risk categories (**Figure 7 – upper rows**). As expected, individuals in the high-risk group experienced the lowest probabilities for events and survival, whereas those in the low-risk group had the most favourable outcomes. When comparing cohorts, ARIC participants consistently showed lower event-free probabilities than those in the UK Biobank.

For example, among individuals in the high-risk group, event-free probability for cardiac events was approximately 92% in the UK Biobank but declined to 75% in ARIC, with nearly twice the number of observed events. In terms of event-free probability across the HyperTrajectories in ARIC and the UK Biobank (**Figure 7 – bottom rows**), events followed similar patterns of incidence across trajectories. For example, ARIC participants in trajectory 1 had the worst probability pattern for cardiac events and trajectory 5 had the highest mortality rates in both datasets with particularly low event-free probabilities for renal diseases in the overall cohorts. In terms of diseases proportions per trajectory, trajectory 2 in both the UK Biobank and ARIC had the highest proportion of metabolic disease, whereas trajectory 4 had the highest proportion of brain outcomes in both datasets.

Computational requirements and training time

Training the cTI model required nearly 5 minutes on a workstation with Intel i7 processor and an RTX 3080 GPU. Once trained, the model is straightforward to apply for prediction and analysis. The *HyperTrajectory* characterisation algorithm requires approximately 10-15 minutes to identify major pathways in the MST graph, with shorter runtimes achievable on

higher-performance workstations. The processing time is dependent on the sample size used and computer specifications.

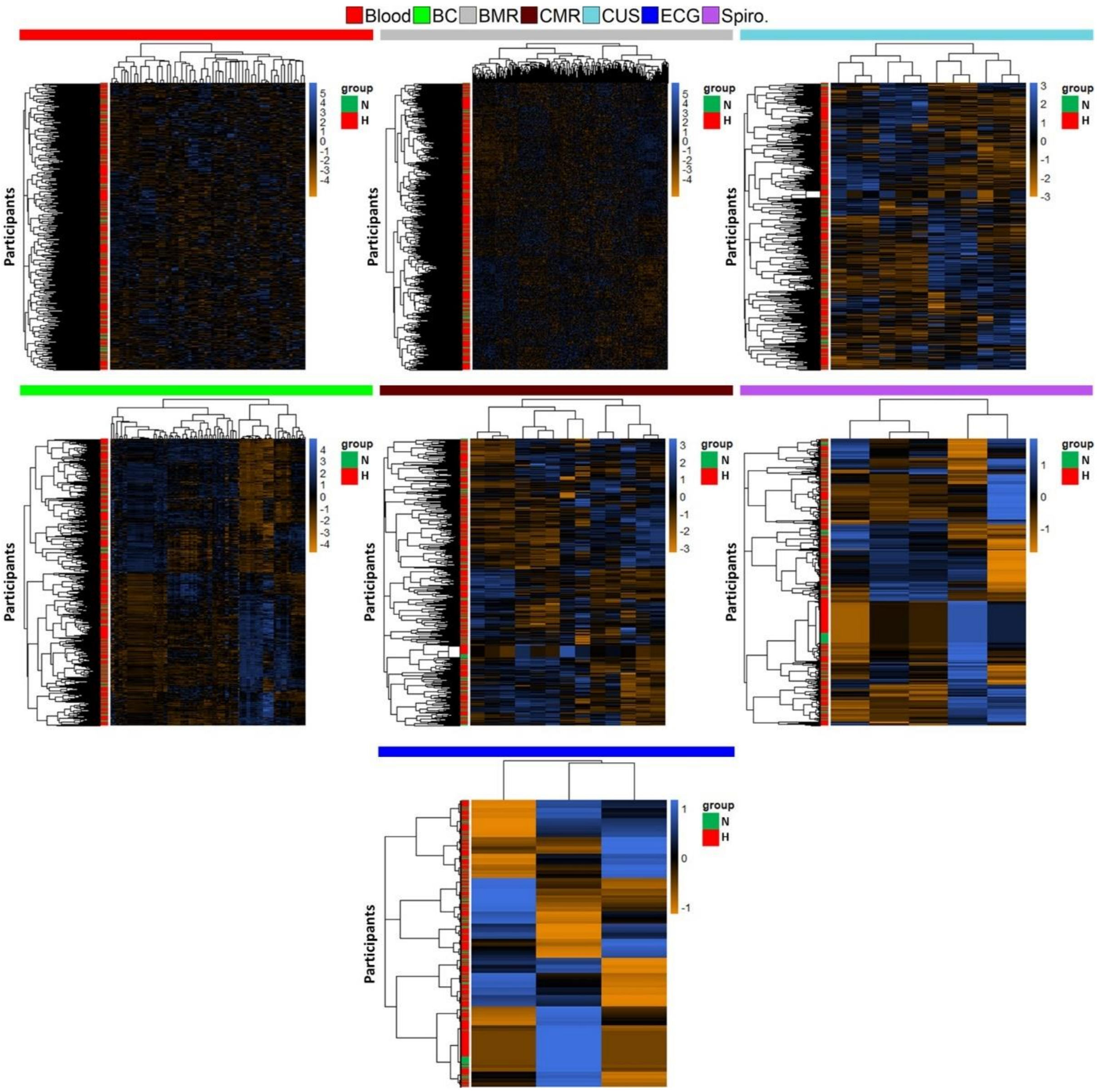

Figure S1. Hierarchical clustering representations of the 566 selected variables in this study grouped by modality. Correlation-based clustering was performed on both rows (participants) and columns (variables) for every modality using the variables selected in this study. The clustering was performed to view the relation between variables in normotensive (N) and hypertensive (H) participants of the overall dataset. Wide color-alike regions indicate similarity between participants or variables. The hierarchical clustering was used to visually analyze the complexity of per-modality features and was not used for feature selection.

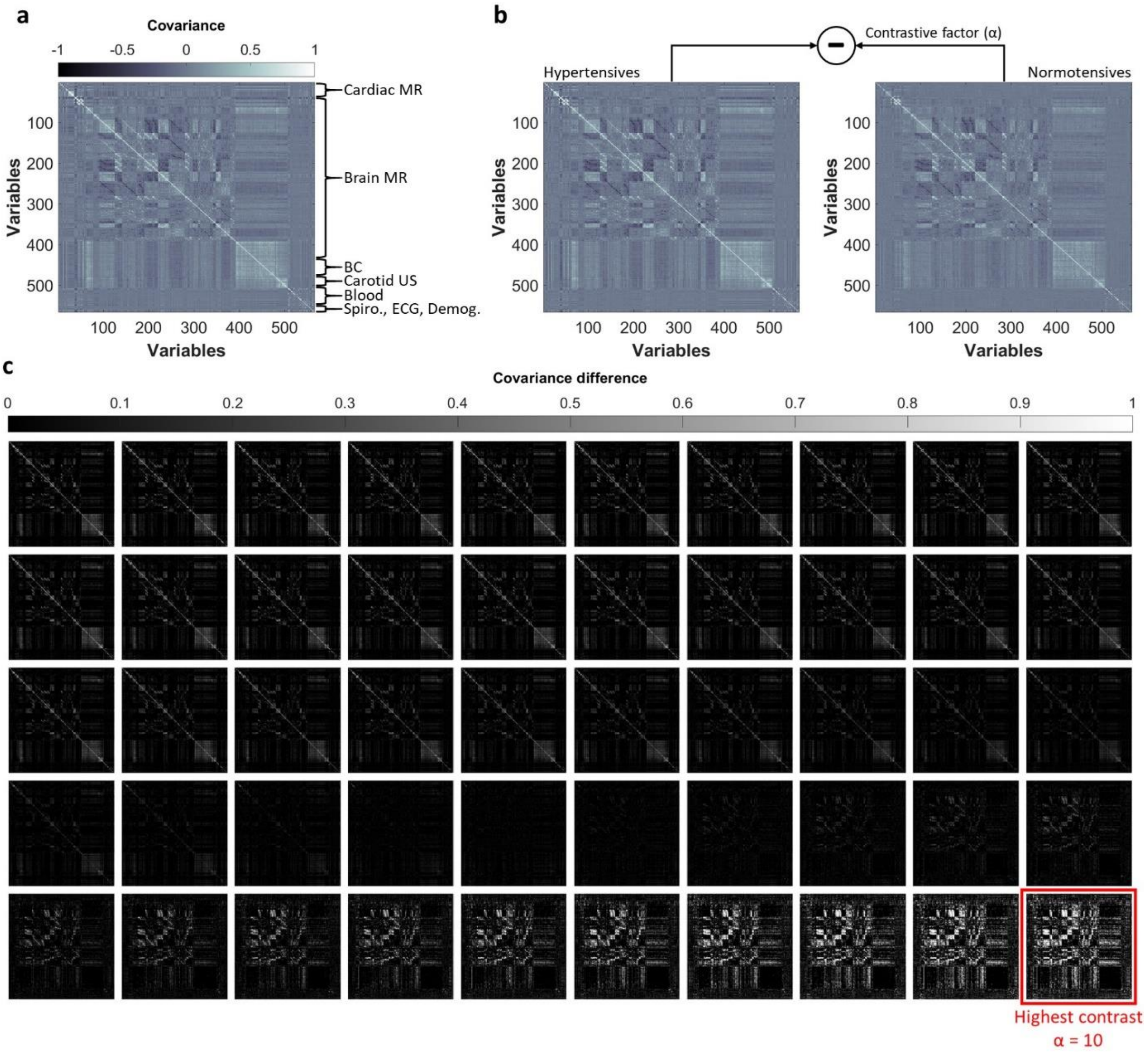

Figure S2. Preparation and selection of the optimal contrastive covariance matrix for the normotensive and hypertensive participants in the overall dataset. a, Covariance representation of the combined set with 566 variables ordered by modality. **b,** Subtraction between the covariance matrices of the hypertensives (left) and normotensives (right). The normotensives covariance matrix is iteratively multiplied by a contrastive factor (α) prior to the subtraction. **c,** Generating 50 contrasts of the resulting subtracted covariance and selecting the optimal factor with the highly contrastive covariance for the contrastive principal components analysis (cPCA).

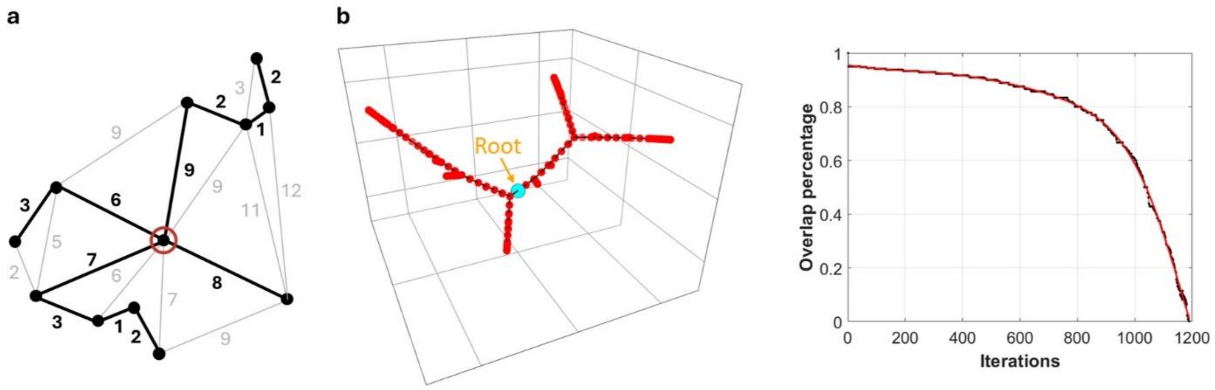

Figure S3. Contrastive trajectory inference (cTI) and construction of the minimum spanning tree (MST). a, cTI defines an MST un-directed graph by connecting all individuals in a way that minimizes the total distance. **b,** The merging of overlapping trajectories reduces redundancy and identifies "clusters" in the dataset, which are then used to define major phenotypes.

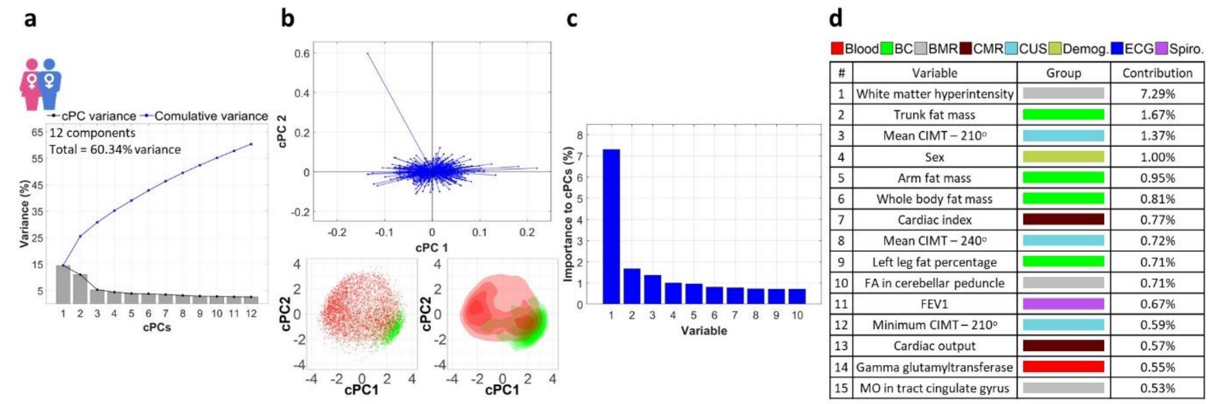

#	Variable	Group	Contribution
1	White matter hyperintensity		7.29%
2	Trunk fat mass		1.67%
3	Mean CIMT – 210°		1.37%
4	Sex		1.00%
5	Arm fat mass		0.95%
6	Whole body fat mass		0.81%
7	Cardiac index		0.77%
8	Mean CIMT – 240°		0.72%
9	Left leg fat percentage		0.71%
10	FA in cerebellar peduncle		0.71%
11	FEV1		0.67%
12	Minimum CIMT – 210°		0.59%
13	Cardiac output		0.57%
14	Gamma glutamyltransferase		0.55%
15	MO in tract cingulate gyrus		0.53%

Figure S4. Interpretation of the modelled contrastive principal components analysis (cPCA)-space. a, Variance explained by the generated cPCs with the corresponding total variance. **b,** Bi-plot representation of the eigen vectors (loadings) showing how strongly each vector influences the first two cPCs. A representation of the first two cPC values is also provided showing the split between normotensives (green) and hypertensives (red). **c,** Bar plot representation of the overall importance (%) of top 10 variables contributing to the generated cPCA space. **d,** Table representation of the top 15 most important variables contributing to the modelled cPCA space.

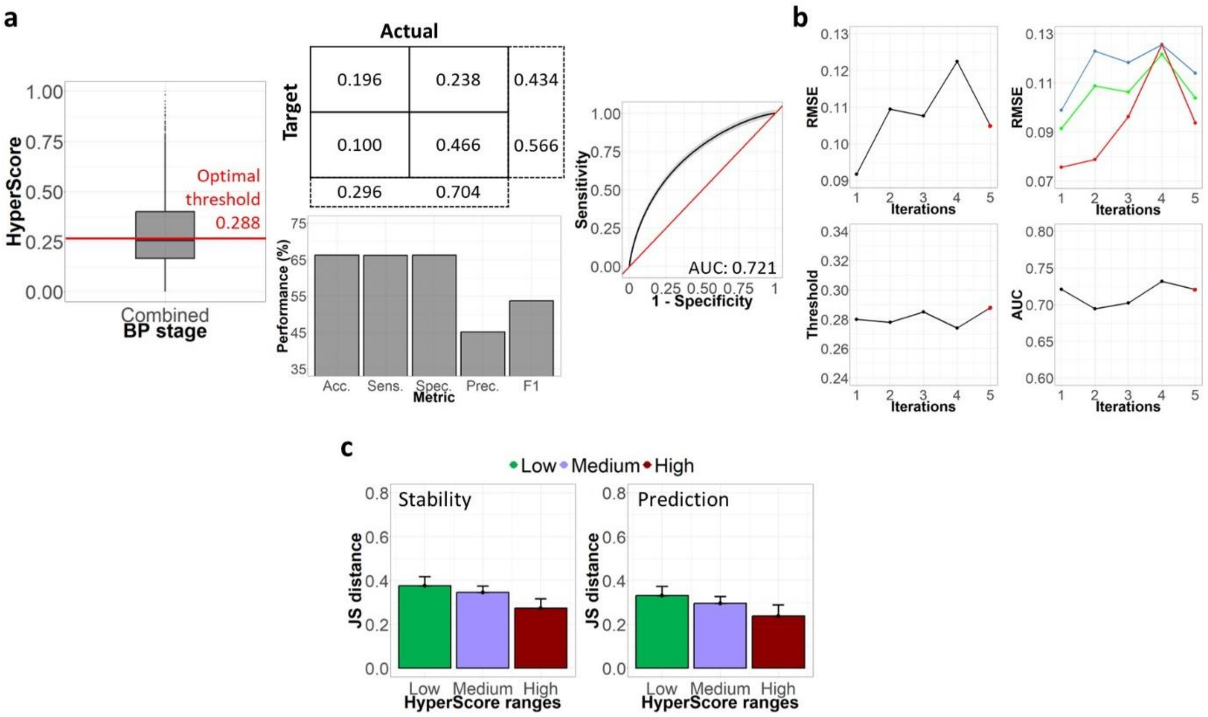

Figure S5. Additional internal validation of the *HyperScore*. a, Validation in absence of ground truth using confidence-based performance estimation (CBPE). *HyperScore* values were used as probabilities towards being hypertensive (0 to 1 scale), and the optimal threshold was calculated to ensure maximum area under the receiver operating characteristics (ROC) curve. The confusion matrix was formed by summation of true/false and positive/negative probabilities relative to the selected threshold. **b,** Data drifting analysis through *HyperScore* prediction error on a testing set after continuously adding data to the trained model on each iteration (top row). The same analysis was applied on the confidence-based metrics (threshold and area under the curve – AUC) when iteratively adding data to the training set. **c,** Jensen-Shannon (JS) distance for stability and prediction when categorizing participants based on *HyperScore*, i.e., low-, medium-, and high-risk.

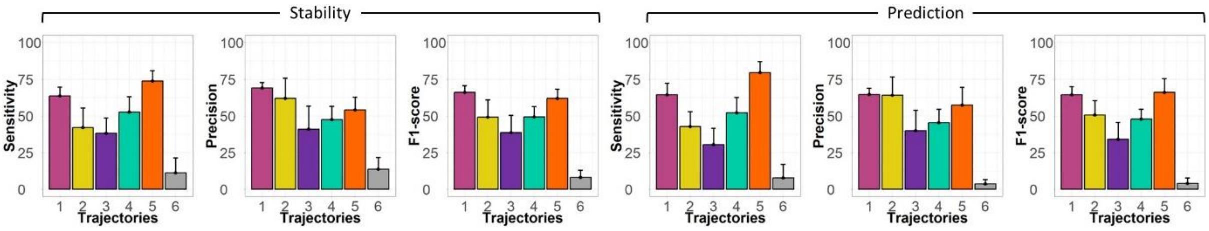

Figure S6. Internal validation performance of the *HyperTrajectory*. Per-trajectory average sensitivity, precision, and F1-score for stability and prediction with the standard deviation (SD) error bar. The ground-truth is selected as the *HyperTrajectory* map of the original full model.

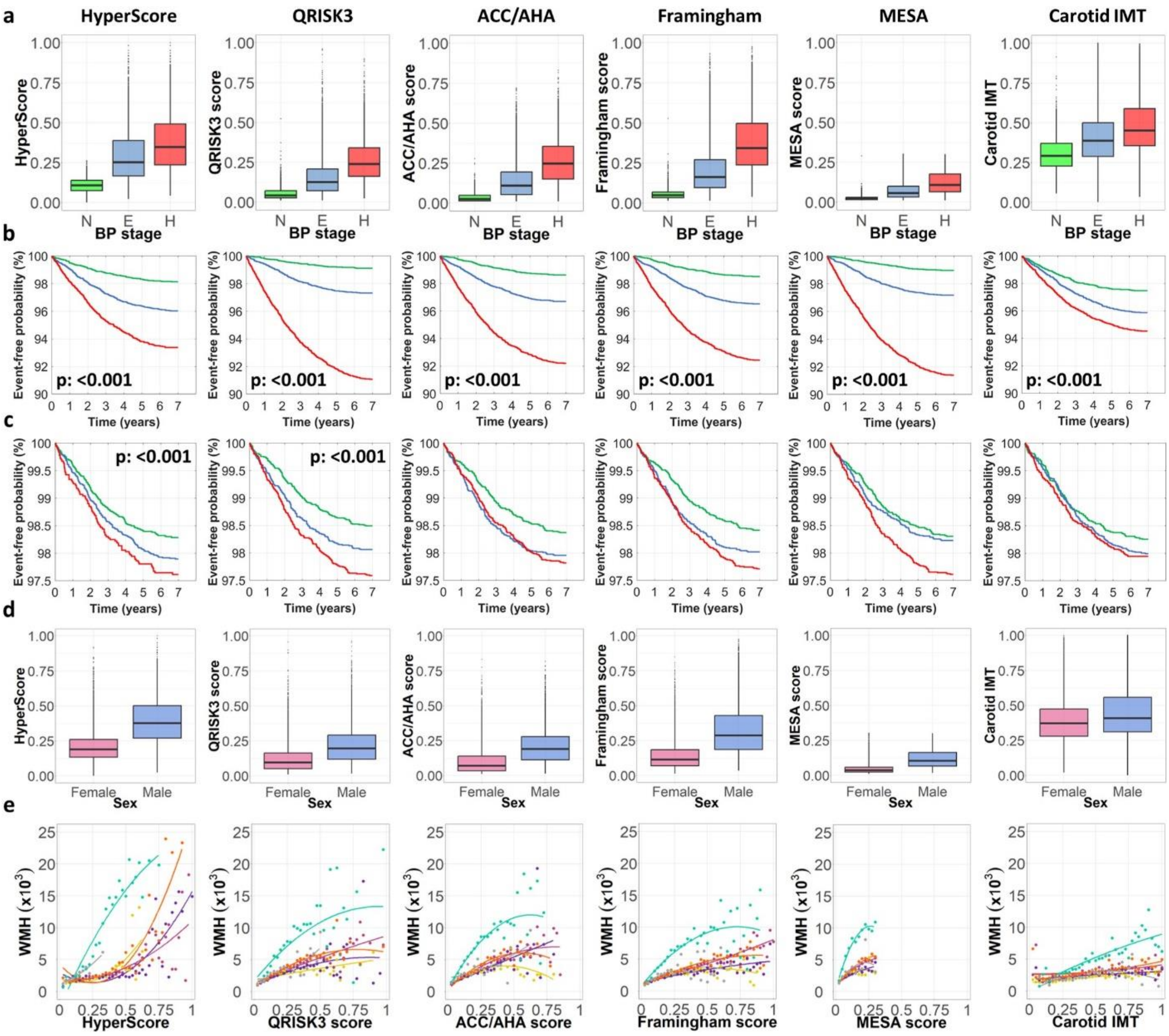

Figure S7. Comparison of *HyperScore* and *HyperTrajectory* relative to conventional and clinically valid scores. a, Scores grouped based on blood pressure stages. **b,** Survival curves of circulatory deaths and diseases when grouping scores into three quantiles based on distribution of scores. **c,** Survival curves for a selected disease, namely liver disorders. **d,** Sex-based analysis of the generated scores with respect to male and female individuals. **e,** A selected example of the phenotypical pattern of progression for white matter hyperintensities with scores at different *HyperTrajectories*. Points within plots represent median value of participants at each 0.025 *HyperScore* step.

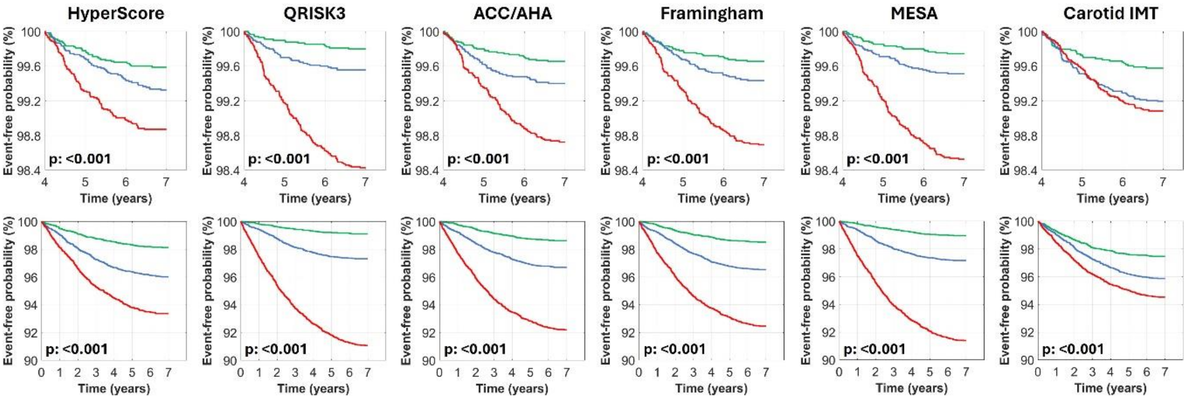

Figure S8. Circulatory diseases and deaths event-free probability for *HyperScore* and other scores with different thresholds. Probabilities were estimated for events happening 4-7 years (upper row) and 0-7 years (bottom row) after the imaging visit. The latter is similar to the results of the overall analysis presented in **Figure S7b**.

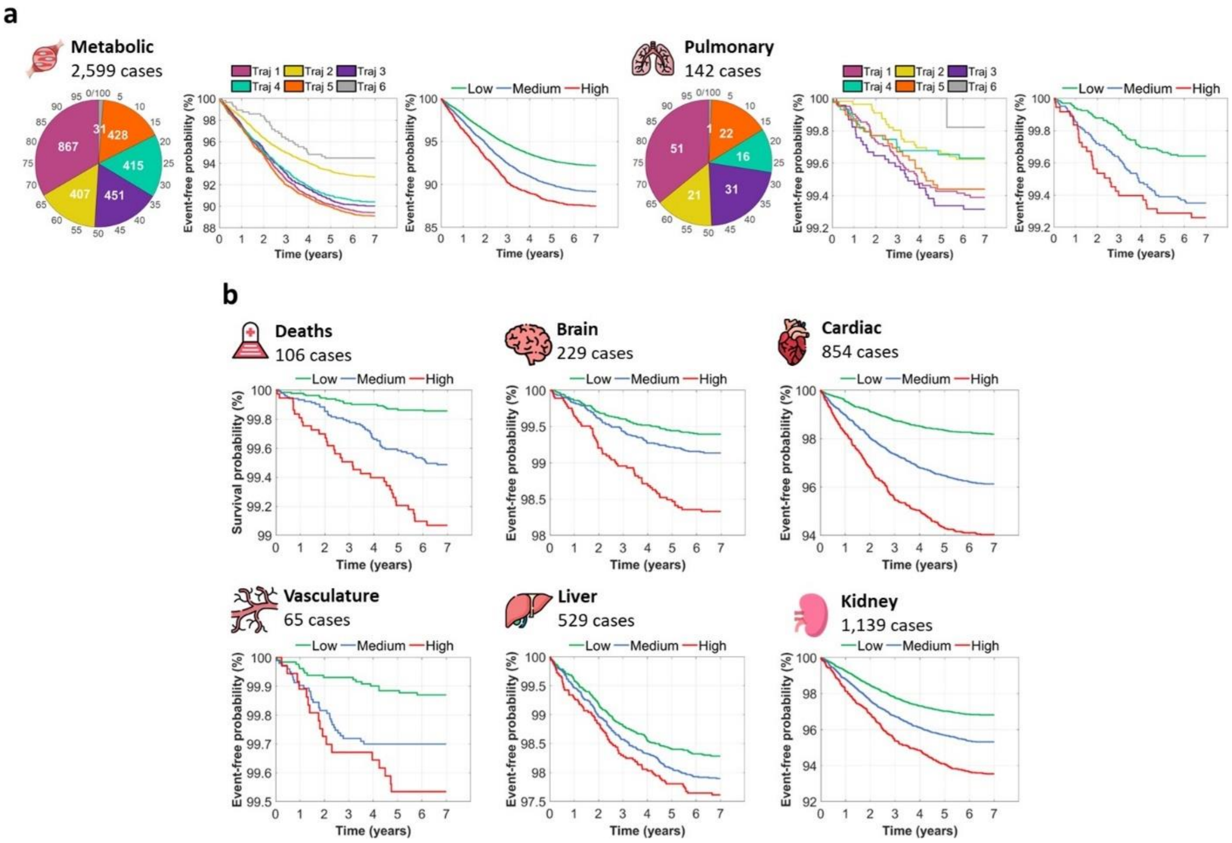

Figure S9. Additional survival analysis on the hypertension progression trajectories and *HyperScore* groups in the overall UK Biobank cohort. a, Metabolic and pulmonary diseases analysis of survival when grouped based on trajectories and based on *HyperScore*. **b,** Survival curves for multiple body organs diseases when grouped based on *HyperScore*.

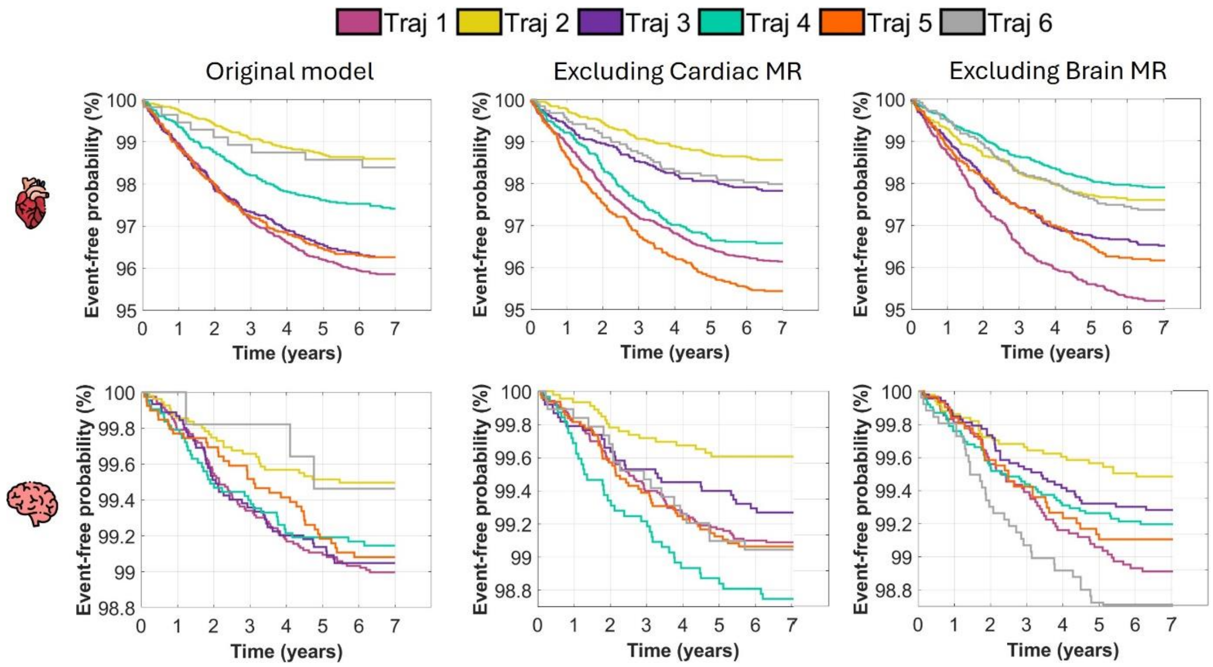

Figure S10. Event-free probability of *HyperTrajectories* after excluding cardiac MR and brain MR variables. Sensitivity analyses were conducted for two outcomes: cardiac disease (upper row) and brain disease (bottom row). Event-free probabilities were additionally estimated using models in which the corresponding imaging modality blocks were excluded before constructing the *HyperTrajectories*.

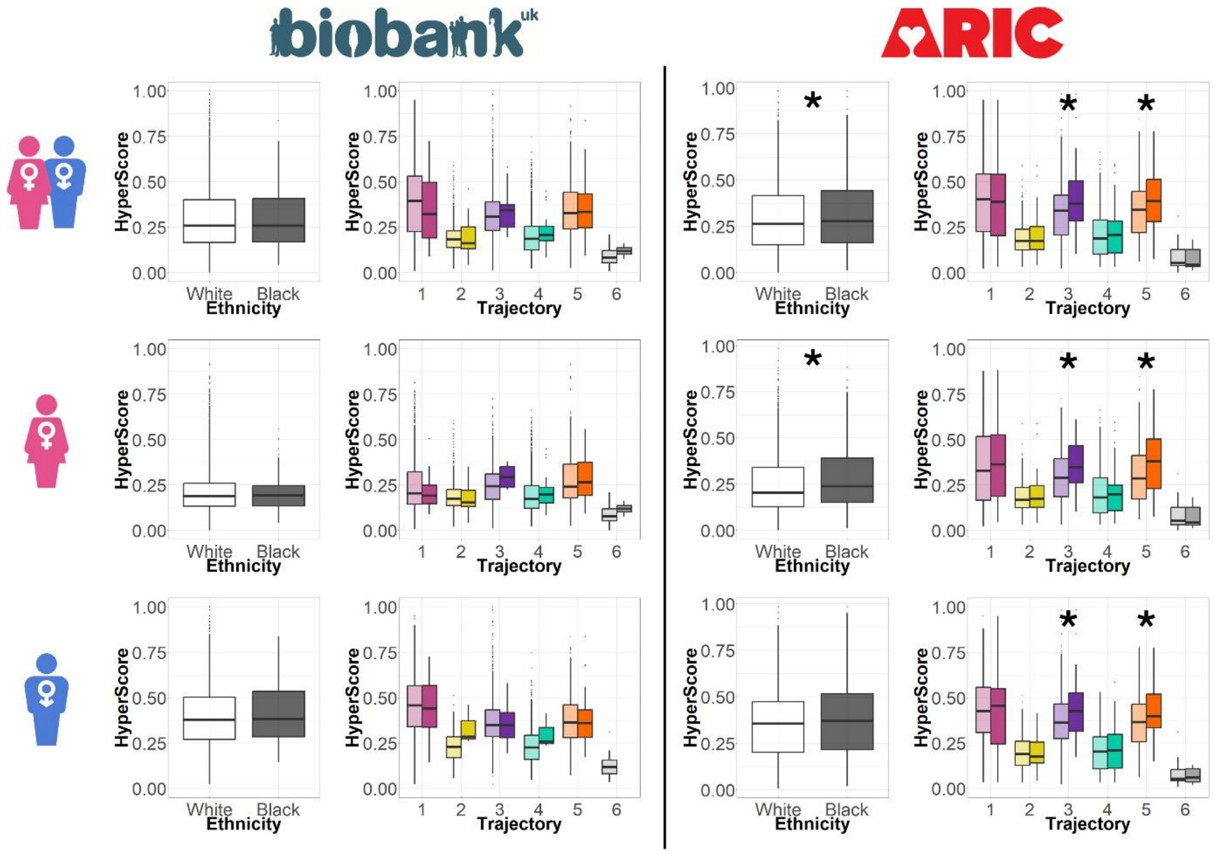

Figure S11. Distribution of *HyperScore* by ethnicity and sex intersections across the UK Biobank and ARIC overall cohorts. Lighter colour in *HyperTrajectory* analysis denotes White ethnicity (left-side box), whereas darker colour indicates Black (right-side box). Star denotes significantly different distributions between both ethnicities in *HyperScore*.

Table S1. Baseline characteristics of the UK Biobank cohort included in this study with male and female analysis.

Variable	Overall (N = 27,099)					Female (N = 14,467 – 53.39%)					Male (N = 12,632 – 46.61%)					***p*-value** (F × M)
	Normotensive (N = 1,386 – 5.12%)	Elevated (N = 21,544 – 79.50%)	Hypertensive (N = 4,169 – 15.38%)	Combined	*p*-value	Normotensive (N = 1,179 – 8.15%)	Elevated (N = 11,385 – 78.70%)	Hypertensive (N = 1,903 – 13.15%)	Combined	*p*-value	Normotensive (N = 207 – 1.64%)	Elevated (N = 10,159 – 80.42%)	Hypertensive (N = 2,266 – 17.94%)	Combined	*p*-value	
**Age,** *years*	58.19±6.59	62.91±7.39	66.84±6.72	63.27±7.48	**<0.001**$^{*o+}$	57.95±6.45	62.54±7.19	66.84±6.50	62.73±7.33	**<0.001**$^{*o+}$	59.56±7.16	63.32±7.59	66.84±6.90	63.89±7.60	**<0.001**$^{*o+}$	**<0.001**^{ne}
**BMI,** *kg/m*^{2}	23.92±3.43	26.26±3.95	27.06±4.00	26.26±3.98	**<0.001**$^{*o+}$	23.82±3.48	25.85±4.22	26.74±4.36	25.80±4.23	**<0.001**$^{*o+}$	24.52±3.02	26.71±3.58	27.31±3.65	26.79±3.60	**<0.001**$^{*o+}$	**<0.001**^{neh}
**Cholesterol level,** *mmol/L*	5.43±1.00	5.74±1.06	5.85±1.11	5.74±1.07	**<0.001**$^{*o+}$	5.43±0.99	5.85±1.05	6.07±1.10	5.85±1.06	**<0.001**$^{*o+}$	5.37±1.01	5.62±1.06	5.66±1.09	5.62±1.07	**<0.001**^{*o}	**<0.001**^{eh}
**Diabetic**, n (%)	11 (0.79%)	1,042 (4.84%)	229 (5.49%)	1,282 (4.73%)	**<0.001**^{*o}	10 (0.85%)	359 (3.15%)	89 (4.68%)	458 (3.17%)	**<0.001**$^{*o+}$	1 (0.48%)	683 (6.72%)	140 (6.18%)	824 (6.52%)	0.681^{*o}	**<0.001**^{eh}
**Hypertension medication**, n (%)<br>------<br>a. Cholesterol medication<br>b. Other<br>c. Unreported	0 (0.00%)<br>------<br>a. 62 (4.47%)<br>b. 133 (9.60%)<br>c. 1,198 (86.44%)	4,392 (20.39%)<br>------<br>a. 4,356 (20.22%)<br>b. 1,053 (4.89%)<br>c. 14,302 (66.39%)	1,431 (34.32%)<br>------<br>a. 1,145 (27.46%)<br>b. 138 (3.31%)<br>c. 2,217 (53.18%)	5,823 (21.49%)<br>------<br>a. 5,563 (20.53%)<br>b. 1,324 (4.89%)<br>c. 17,717 (65.38%)	**<0.001**$^{*o+}$	0 (0.00%)<br>------<br>a. 51 (4.33%)<br>b. 133 (11.28%)<br>c. 1,002 (84.99%)	1,832 (16.09%)<br>------<br>a. 1,502 (13.19%)<br>b. 973 (8.55%)<br>c. 8,014 (70.39%)	578 (30.37%)<br>------<br>a. 376 (19.76%)<br>b. 122 (6.41%)<br>c. 1,093 (57.44%)	2,410 (16.66%)<br>------<br>a. 1,929 (13.33%)<br>b. 1,228 (8.49%)<br>c. 10,109 (69.88%)	**<0.001**$^{*o+}$	0 (0.00%)<br>------<br>a. 11 (5.31%)<br>b. 0 (0.00%)<br>c. 196 (94.69%)	2,560 (25.20%)<br>------<br>a. 2,854 (28.09%)<br>b. 80 (0.79%)<br>c. 6,288 (61.90%)	853 (37.64%)<br>------<br>a. 769 (33.94%)<br>b. 16 (0.71%)<br>c. 1,124 (49.60%)	3,413 (27.02%)<br>------<br>a. 3,634 (28.77%)<br>b. 96 (0.76%)<br>c. 7,608 (60.23%)	**<0.001**$^{*o+}$	**<0.001**^{eh}
**White ethnicity**, n (%)<br>------<br>a. Asian or Asian British<br>b. Black or black British<br>c. Chinese<br>d. Other mixed<br>e. Unreported	1,327 (95.74%)<br>------<br>a. 17 (1.23%)<br>b. 8 (0.58%)<br>c. 6 (0.43%)<br>d. 10 (0.72%)<br>e. 18 (1.30%)	20,861 (96.83%)<br>------<br>a. 210 (0.97%)<br>b. 119 (0.55%)<br>c. 63 (0.29%)<br>d. 100 (0.46%)<br>e. 191 (0.89%)	4,066 (97.53%)<br>------<br>a. 39 (0.94%)<br>b. 23 (0.55%)<br>c. 11 (0.26%)<br>d. 8 (0.19%)<br>e. 22 (0.53%)	26,254 (96.88%)<br>------<br>a. 266 (0.98%)<br>b. 150 (0.55%)<br>c. 80 (0.30%)<br>d. 118 (0.44%)<br>e. 231 (0.85%)	**<0.001**$^{*o+}$	1,134 (96.18%)<br>------<br>a. 10 (0.85%)<br>b. 6 (0.51%)<br>c. 5 (0.42%)<br>d. 8 (0.68%)<br>e. 16 (1.36%)	11,047 (97.03%)<br>------<br>a. 73 (0.64%)<br>b. 64 (0.56%)<br>c. 37 (0.32%)<br>d. 63 (0.55%)<br>e. 101 (0.89%)	1,862 (97.85%)<br>------<br>a. 12 (0.63%)<br>b. 12 (0.63%)<br>c. 6 (0.32%)<br>d. 6 (0.32%)<br>e. 5 (0.26%)	14,043 (97.07%)<br>------<br>a. 95 (0.66%)<br>b. 82 (0.57%)<br>c. 48 (0.33%)<br>d. 77 (0.53%)<br>e. 122 (0.84%)	**0.007**$^{o+}$	193 (93.24%)<br>------<br>a. 7 (3.38%)<br>b. 2 (0.97%)<br>c. 1 (0.48%)<br>d. 2 (0.97%)<br>e. 2 (0.97%)	9,814 (96.60%)<br>------<br>a. 137 (1.35%)<br>b. 55 (0.54%)<br>c. 26 (0.26%)<br>d. 37 (0.36%)<br>e. 90 (0.89%)	2,204 (97.26%)<br>------<br>a. 27 (1.19 %)<br>b. 11 (0.49%)<br>c. 5 (0.22%)<br>d. 2 (0.09%)<br>e. 17 (0.75%)	12,211 (96.67%)<br>------<br>a. 171 (1.35%)<br>b. 68 (0.54%)<br>c. 32 (0.25%)<br>d. 41 (0.32%)<br>e. 109 (0.86%)	**0.013**^{*o}	0.058
**Currently smoker**, n (%)<br>------<br>a. Previous<br>b. Never<br>c. Unreported	55 (3.97%)<br>------<br>a. 416 (30.01%)<br>b. 906 (65.37%)<br>c. 9 (0.65%)	767 (3.56%)<br>------<br>a. 7,001 (32.50%)<br>b. 13,628 (63.26%)<br>c. 148 (0.69%)	95 (2.28%)<br>------<br>a. 7,001 (32.50%)<br>b. 2,499 (59.94%)<br>c. 47 (1.13%)	917 (3.38%)<br>------<br>a. 1,528 (36.65%)<br>b. 17,033 (62.85%)<br>c. 204 (0.75%)	**<0.001**$^{o+}$	40 (3.39%)<br>------<br>a. 361 (30.62%)<br>b. 769 (65.22%)<br>c. 9 (0.76%)	339 (2.98%)<br>------<br>a. 3,472 (30.50%)<br>b. 7,488 (65.77%)<br>c. 86 (0.76%)	32 (1.68%)<br>------<br>a. 610 (32.05%)<br>b. 1,236 (64.95%)<br>c. 25 (1.31%)	411 (2.84%)<br>------<br>a. 4,443 (30.71%)<br>b. 9,493 (65.62%)<br>c. 120 (0.83%)	**0.002**$^{o+}$	15 (7.25%)<br>------<br>a. 55 (26.57%)<br>b. 137 (66.18%)<br>c. 0 (0.00%)	428 (4.21%)<br>------<br>a. 3,529 (34.74%)<br>b. 6,140 (60.44%)<br>c. 62 (0.61%)	63 (2.78%)<br>------<br>a. 918 (40.51%)<br>b. 1,263 (55.74%)<br>c. 22 (0.97%)	506 (4.01%)<br>------<br>a. 4,502 (35.64%)<br>b. 7,540 (59.69%)<br>c. 84 (0.66%)	**<0.001**$^{*o+}$	**<0.001**^{nch}
**Heavy alcohol drinker**, n (%)<br>------<br>a. Moderate alcohol drinker<br>b. Unreported	926 (66.81%)<br>------<br>a. 454 (32.76%)<br>b. 6 (0.43%)	15,421 (71.58%)<br>------<br>a. 6,021 (27.95%)<br>b. 102 (0.47%)	3,034 (72.78%)<br>------<br>a. 1,103 (26.46%)<br>b. 32 (0.77%)	19,381 (71.52%)<br>------<br>a. 7,578 (27.96%)<br>b. 140 (0.52%)	**<0.001**^{*o}	765 (64.89%)<br>------<br>a. 408 (34.61 %)<br>b. 6 (0.51%)	7,482 (65.72%)<br>------<br>a. 3,852 (33.83%)<br>b. 51 (0.45%)	1,224 (64.32%)<br>------<br>a. 660 (34.68%)<br>b. 19 (1.00%)	9,471 (65.47%)<br>------<br>a. 4,920 (34.01%)<br>b. 76 (0.53%)	0.568	161 (77.78%)<br>------<br>a. 46 (22.22%)<br>b. 0 (0.00%)	7,939 (78.15%)<br>------<br>a. 2,169 (21.35%)<br>b. 51 (0.50%)	1,810 (79.88%)<br>------<br>a. 443 (19.55%)<br>b. 13 (0.57%)	9,910 (78.45%)<br>------<br>a. 2,658 (21.04%)<br>b. 64 (0.51%)	0.076	**<0.001**^{nch}

Continuous variables are represented as Mean±SD. Categorical variables are represented as n (%). Bold *p*-values show statistically significant differences ($p < 0.05$) between all three blood pressure groups using one-way analysis of variance (ANOVA) for continuous variables. Categorical variables were statistically analyzed by fitting a generalized linear model. BMI = Body mass index; *: significance between normotensive and

elevated; o: significance between normotensive and hypertensive; +: significance between elevated and hypertensive; n: significance between normotensive female and male; e: significance between elevated female and male; h: significance between hypertensive female and male.

Table S2. Characteristics of the *HyperTrajectory* in the overall UK Biobank cohort.

HyperTrajectory	1 (N = 8,187)	2 (N = 5,579)	3 (N = 4,523)	4 (N = 4,326)	5 (N = 3,921)	6 (N = 561)
HyperScore	0.39±0.19	0.19±0.07	0.33±0.14	0.21±0.11	0.35±0.15	0.09±0.05
**Demographic information**						
**Female**	2,572 (31.42%)	5,053 (90.57%)	1,807 (39.95%)	3,425 (79.17%)	1,109 (28.28%)	500 (89.13%)
**Age, *years***	64.44±7.51	61.53±6.87	62.72±7.51	64.07±7.49	63.34±7.66	61.32±7.34
**SBP, *mmHg***	142.32±19.21	136.20±19.91	140.19±18.94	139.38±20.16	142.21±18.53	133.31±19.21
**DBP, *mmHg***	79.51±10.40	76.82±10.28	79.36±10.41	77.88±10.54	79.94±10.29	76.57±10.15
**PP, *mmHg***	62.81±15.54	59.38±15.91	60.84±15.11	61.50±16.29	62.27±15.02	56.73±15.06
**MAP, *mmHg***	100.45±11.89	96.62±12.09	99.63±11.88	98.38±12.27	100.70±11.61	95.49±11.89
**Current smoker**	296 (3.62%)	154 (2.76%)	157 (3.47%)	124 (2.87%)	173 (4.41%)	14 (2.5%)
**Diabetic**	587 (7.17%)	108 (1.94%)	218 (4.82%)	151 (3.49%)	207 (5.28%)	12 (2.14%)
**Heavy alcohol drinker**	2,135 (26.08%)	1,874 (33.59%)	1,198 (26.49%)	1,335 (30.86%)	984 (25.10%)	194 (34.58%)
**Hypertension Medication**	2,129 (26.00%)	721 (12.92%)	1,018 (22.51%)	907 (20.97%)	968 (24.69%)	83 (14.80%)
**Cholesterol Medication**	2,162 (26.41%)	630 (11.29%)	998 (22.07%)	785 (18.15%)	913 (23.28%)	75 (13.37%)
**Imaging/non-imaging measurements**						
**WMH, *x10³ mm³***	4.29±4.00	2.611±2.21	3.34±3.24	6.37±5.83	4.25±4.20	2.81±2.19
**GM, *x10⁴ mm³***	78.28±4.60	81.52±4.25	79.30±4.41	79.93±4.56	78.77±4.50	81.32±4.48
**CO, *L/min***	4.808±1.126	4.403±0.999	4.81±1.104	4.412±1.018	4.934±1.109	4.343±0.979
**CI, *L/min/m²***	2.517±0.523	2.521±0.503	2.53±0.505	2.475±0.498	2.551±0.518	2.48±0.498
**COp, *L/min***	6.82±2.28	7.53±2.62	6.73±2.34	7.44±2.58	6.73±2.24	7.33±2.46
**CIp, *L/min/m²***	3.62±1.37	4.39±1.62	3.60±1.42	4.24±1.59	3.52±1.31	4.20±1.50
**LVEF, *%***	55.40±5.75	57.04±5.32	55.70±5.64	56.64±5.40	55.33±5.84	57.10±5.29
**VR, *bpm***	61.47±10.19	61.00±9.37	60.97±9.91	61.44±9.67	61.05±9.90	60.83±9.75
**BMI, *kg/m²***	26.76±3.96	25.43±3.90	26.53±3.94	25.83±4.09	26.69±3.78	25.52±4.19
**BFM, *kg***	23.08±7.88	23.68±7.66	23.11±8.06	23.95±8.33	22.67±7.53	23.98±8.27
**Cholesterol, *mmol/L***	5.63±1.06	5.90±1.02	5.70±1.03	5.77±1.03	5.67±1.03	5.69±1.00
**LDL, *mmol/L***	3.53±0.81	3.65±0.79	3.59±0.79	3.57±0.79	3.58±0.80	3.49±0.78
**HDL, *mmol/L***	1.39±0.34	1.61±0.35	1.42±0.34	1.57±0.35	1.38±0.33	1.60±0.35
**Triglycerides, *mmol/L***	1.69±0.85	1.36±0.67	1.65±0.79	1.42±0.70	1.70±0.82	1.30±0.65
**HbA1c, *mmol/mol***	34.91±3.88	34.11±3.48	34.51±3.71	34.44±3.65	34.63±3.70	34.15±3.73
**GLC, *mmol/L***	4.94±0.64	4.88±0.59	4.89±0.60	4.94±0.60	4.93±0.60	4.88±0.59
**ALT, *U/L***	23.83±10.41	18.20±7.85	23.46±10.34	19.65±8.50	23.71±9.92	18.60±7.73
**GGT, *U/L***	34.62±21.75	22.98±13.98	33.02±21.05	25.21±15.53	34.74±21.30	23.29±14.26
**TP, *g/L***	72.24±3.76	72.01±3.78	72.28±3.83	72.09±3.75	72.31±3.82	72.15±3.78
**CRTN, *µmol/L***	75.81±13.25	65.23±10.73	74.07±13.11	67.15±11.65	75.97±12.70	65.28±10.38
**CysC, *mg/L***	0.89±0.12	0.83±0.11	0.87±0.11	0.85±0.12	0.88±0.12	0.83±0.11
**WBC, *x10⁹ cells/L***	6.60±1.54	6.42±1.49	6.52±1.55	6.39±1.51	6.58±1.51	6.48±1.56
**RTIC, *x10¹² cells/L***	0.060±0.024	0.052±0.021	0.060±0.024	0.054±0.022	0.061±0.023	0.053±0.022
**Adverse outcomes**						
**Death** (Circulatory diseases)	43 (2.18%)	6 (0.75%)	20 (2.00%)	15 (1.73%)	21 (2.23%)	1 (1.32%)
**Cardiac**	339 (17.16%)	78 (9.73%)	169 (16.90%)	112 (12.90%)	147 (15.62%)	9 (11.84%)
**Brain**	82 (4.15%)	28 (3.49%)	43 (4.30%)	37 (4.26%)	36 (3.83%)	3 (3.95%)
**Vasculature**	24 (1.22%)	8 (1.00%)	14 (1.40%)	7 (0.81%)	12 (1.28%)	0 (0.00%)
**Pulmonary**	51 (2.58%)	21 (2.62%)	31 (3.10%)	16 (1.84%)	22 (2.34%)	1 (1.32%)
**Liver**	164 (8.30%)	93 (11.60%)	99 (9.90%)	85 (9.79%)	75 (7.97%)	13 (17.11%)
**Kidney**	406 (20.55%)	161 (20.08%)	173 (17.30%)	181 (20.85%)	200 (21.25%)	18 (23.68%)
**Metabolic**	867 (43.88%)	407 (50.75%)	451 (45.10%)	415 (47.81%)	428 (45.48%)	31 (40.79%)
**Overall**	1,976 (24.14%)	802 (14.38%)	1,000 (22.11%)	868 (20.07%)	941 (24.00%)	76 (13.55%)

Continuous variables are represented as Mean±SD. Categorical variables are represented as n (%). The percentage of organ-specific outcomes is based on the overall number of outcomes.

The percentage of the overall outcomes is based on the total number of participants. SBP = Systolic blood pressure; DBP = Diastolic blood pressure; PP = Pulse pressure; MAP = Mean arterial pressure; WMH = White matter hyperintensity; GM = Gray matter volume; CO = Cardiac output; CIp = Cardiac index; COp = Cardiac output during pulse wave analysis (PWA); CIp = Cardiac index during PWA; LVEF = Left ventricular ejection fraction; VR = Ventricular rate; BMI = Body mass index; BFM = Body fat mass; LDL = Low-density lipoprotein; HDL = High-density lipoprotein; HbA1c = Glycated hemoglobin; GLC = Glucose; ALT = Alanine aminotransferase; GGT = Gamma glutamyltransferase; TP = Total protein; CRTN = Creatinine; CysC = Cystatin C; WBC = White blood cells count; RTIC = Reticulocytes count.

Table S3. Characteristics of the *HyperTrajectory* in the advanced disease state (top 5% *HyperScores*) in the UK Biobank cohort.

HyperTrajectory	**1** **(N = 410)**	**2** **(N = 279)**	**3** **(N = 227)**	**4** **(N = 217)**	**5** **(N = 197)**	**6** **(N = 29)**
**HyperScore**	0.75±0.06	0.37±0.07	0.70±0.10	0.52±0.05	0.70±0.06	0.19±0.01
**Demographic information**						
**Female**	32 (7.81%)	212 (75.99%)	15 (6.61%)	149 (68.66%)	26 (13.20%)	24 (83.76%)
**Age, *years***	67.42±7.06	63.85±6.68	66.74±7.32	68.53±6.01	69.32±5.80	64.07±8.61
**SBP, *mmHg***	150.78±20.42	149.44±23.97	150.48±20.02	147.63±18.34	151.01±19.08	146.31±23.13
**DBP, *mmHg***	82.25±10.23	81.46±11.49	83.21±10.99	79.64±9.98	81.09±10.59	82.03±10.81
**PP, *mmHg***	68.52±17.07	67.98±20.03	67.27±16.08	67.99±16.13	69.92±15.49	64.28±20.08
**MAP, *mmHg***	105.10±12.00	104.12±13.80	105.63±12.51	102.30±10.98	104.40±11.95	103.46±12.91
**Current smoker**	16 (3.90%)	7 (2.51%)	3 (1.32%)	7 (3.23%)	6 (3.05%)	0 (0.00%)
**Diabetic**	61 (14.88%)	11 (3.94%)	25 (11.01%)	8 (3.69%)	17 (8.63%)	0 (0.00%)
**Heavy alcohol drinker**	321 (78.29%)	194 (69.53%)	178 (78.41%)	142 (65.44%)	154 (78.17%)	23 (79.31%)
**Hypertension Medication**	166 (40.49%)	62 (22.22%)	100 (44.05%)	74 (34.10%)	78 (39.59%)	24 (24.14%)
**Cholesterol Medication**	153 (37.32%)	51 (18.28%)	102 (44.93%)	68 (31.34%)	78 (39.59%)	2 (6.90%)
**Imaging/non-imaging measurements**						
**WMH, *$x10^3$ mm^3***	9.57±6.73	3.74±3.31	8.29±6.88	17.16±4.85	10.94±7.30	5.12±3.69
**GM, *$x10^4$ mm^3***	75.92±39.97	80.35±4.37	76.26±4.38	77.63±3.90	75.55±4.10	79.84±4.78
**CO, *L/min***	4.97±1.16	4.50±1.21	5.15±1.09	4.52±1.09	4.73±0.98	4.69±1.34
**CI, *$L/min/m^2$***	2.46±0.53	2.49±0.58	2.57±0.54	2.49±0.53	2.42±0.50	2.57±0.59
**COp, *L/min***	6.94±2.14	8.23±2.80	7.11±2.02	8.06±2.68	6.97±2.16	8.60±2.23
**CIp, *$L/min/m^2$***	3.47±1.21	4.64±1.73	3.57±1.12	4.55±1.76	3.61±1.27	4.61±1.03
**LVEF, *%***	54.17±5.98	56.97±6.12	55.23±5.56	56.62±6.09	54.55±5.75	56.96±5.97
**VR, *bpm***	63.00±10.32	61.53±9.53	64.63±10.57	62.33±9.14	60.76±10.53	61.96±9.93
**BMI, *kg/m^2***	28.47±3.79	26.14±4.44	28.02±3.95	26.52±4.32	27.28±3.60	28.17±4.24
**BFM, *kg***	25.21±7.54	22.76±7.17	23.86±8.07	24.50±8.57	23.43±6.94	27.82±8.38
**Cholesterol, *mmol/L***	5.49±1.07	5.97±1.08	5.48±1.06	5.83±1.08	5.58±1.02	6.10±0.97
**LDL, *mmol/L***	3.45±0.79	3.71±0.82	3.48±0.82	3.61±0.80	3.52±0.81	3.83±0.70
**HDL, *mmol/L***	1.24±0.29	1.54±0.41	1.27±0.30	1.53±0.39	1.34±0.31	1.56±0.43
**Triglycerides, *mmol/L***	2.04±0.97	1.54±0.77	1.85±0.79	1.54±0.76	1.92±0.96	1.51±0.58
**HbA1c, *mmol/mol***	36.01±4.05	35.18±3.54	35.27±4.16	35.26±3.61	35.66±3.94	34.46±4.29
**GLC, *mmol/L***	5.04±0.62	4.99±0.63	5.02±0.63	5.02±0.64	4.95±0.55	4.85±0.33
**ALT, *U/L***	28.34±11.52	19.83±8.38	29.20±11.76	20.11±8.10	24.93±10.32	22.37±10.12
**GGT, *U/L***	44.04±25.11	27.19±15.81	44.00±25.11	28.61±18.02	40.78±24.62	32.29±25.61
**TP, *g/L***	72.33±3.68	71.87±3.73	73.31±3.78	71.64±3.62	72.05±3.67	73.94±4.11
**CRTN, *µmol/L***	81.46±12.54	67.51±12.63	80.42±11.99	68.25±14.03	79.79±12.58	64.70±10.74
**CysC, *mg/L***	0.93±0.12	0.86±0.12	0.92±0.12	0.87±0.11	0.92±0.12	0.85±0.10
**WBC, *$x10^9$ cells/L***	6.85±1.58	6.68±1.55	6.91±1.53	6.46±1.55	6.76±1.55	7.53±1.91
**RTIC, *$x10^{12}$ cells/L***	0.068±0.025	0.057±0.025	0.070±0.026	0.056±0.022	0.067±0.026	0.065±0.017
**Adverse outcomes**						
**Death** (Circulatory diseases)	4 (3.42%)	0 (0.00%)	**4 (3.51%)**	2 (3.03%)	2 (2.56%)	0 (0.00%)
**Cardiac**	16 (13.68%)	9 (14.29%)	20 (17.54%)	12 (18.18%)	**15 (19.23%)**	0 (0.00%)
**Brain**	9 (7.69%)	1 (1.59%)	9 (7.90%)	**6 (9.09%)**	3 (3.84%)	1 (33.33%)
**Vasculature**	2 (1.71%)	**2 (3.18%)**	2 (1.75%)	1 (1.52%)	2 (2.56%)	0 (0.00%)
**Pulmonary**	**4 (3.42%)**	1 (1.59%)	2 (1.75%)	2 (3.03%)	2 (2.56%)	0 (0.00%)
**Liver**	6 (5.13%)	**8 (12.70%)**	9 (7.90%)	7 (10.61%)	8 (10.26%)	1 (33.33%)
**Kidney**	28 (23.93%)	15 (23.81%)	20 (17.54%)	13 (19.70%)	**19 (24.36%)**	1 (33.33%)
**Metabolic**	48 (41.03%)	**27 (42.86%)**	48 (42.11%)	23 (34.85%)	27 (34.62%)	0 (0.00%)
**Overall**	117 (28.54%)	63 (22.58%)	**114 (50.22%)**	66 (30.42%)	78 (39.59%)	3 (10.35%)

Continuous variables are represented as Mean±SD. Categorical variables are represented as n (%). The percentage of organ-specific outcomes is based on the overall number of outcomes.

The percentage of the overall outcomes is based on the total number of participants. Bold indicates the trajectory with highest number of outcomes if enough outcomes occur. SBP = Systolic blood pressure; DBP = Diastolic blood pressure; PP = Pulse pressure; MAP = Mean arterial pressure; WMH = White matter hyperintensity; GM = Gray matter volume; CO = Cardiac output; CIp = Cardiac index; COp = Cardiac output during pulse wave analysis (PWA); CIp = Cardiac index during PWA; LVEF = Left ventricular ejection fraction; VR = Ventricular rate; BMI = Body mass index; BFM = Body fat mass; LDL = Low-density lipoprotein; HDL = High-density lipoprotein; HbA1c = Glycated hemoglobin; GLC = Glucose; ALT = Alanine aminotransferase; GGT = Gamma glutamyltransferase; TP = Total protein; CRTN = Creatinine; CysC = Cystatin C; WBC = White blood cells count; RTIC = Reticulocytes count.

Table S4. Examples from the UK Biobank dataset showing *HyperScores*, *HyperTrajectories*, demographic information, imaging/non-imaging measurements, and adverse outcomes.

**HyperTrajectory**	**1**	**2**	**3**	**4**	**5**	**6**	**Optimal ranges**
**HyperScore**	0.743	0.523	0.917	0.570	0.777	0.106	
**Demographic information**							
**Sex**	Male	Female	Male	Female	Male	Female	
**Age**, *years*	72	72	73	72	71	50	
**SBP**, *mmHg*	161	165	143	133	151	130	
**DBP**, *mmHg*	71	83	92	67	98	82	
**PP**, *mmHg*	90	82	51	66	53	48	
**MAP**, *mmHg*	101	110	109	89	116	98	
**Current smoker**	No	Yes	Yes	No	No	Yes	
**Diabetic**	No	Yes	No	Yes	No	No	
**Heavy alcohol drinker**	Heavy	Moderate	Heavy	Moderate	Heavy	Heavy	
**Medication**	Cholesterol	Cholesterol Hypertension	-	-	Cholesterol Hypertension	-	
**Imaging/non-imaging measurements**							
**WMH**, *x10^3 mm^3*	2.47	6.72	**17.02**	17.66	24.03	2.28	Desired: Lower
**GM**, *x10^4 mm^3*	85.64	76.13	**69.13**	70.27	74.86	83.49	Desired: Higher
**COp**, *L/min*	**8.26**	**10.46**	8.6	8.08	6.36	7.05	5–6
**CIp**, *L/min/m^2*	**4.12**	**5.1**	4.19	4.71	3.13	3.6	2.5–3.5
**LVEF**, *%*	**43**	63	53	58	54	47	>50
**VR**, *bpm*	62	**80**	57	63	63	74	60-80
**BMI**, *kg/m^2*	27.9	**39.2**	26.1	28.6	25	**32**	20–30
**BFM**, *kg*	27	**44.3**	26.4	28.2	25.6	**36.3**	15–30
**HDL**, *mmol/L*	0.952	0.95	1.323	**1.143**	1.343	1.646	>1.2 (female)
**GLC**, *mmol/L*	5.495	6.707	5.57	**5.596**	4.627	5.025	3.9–5.5
**ALT**, *U/L*	22.73	20.6	24.92	17.1	17.32	**33.44**	4–36
**GGT**, *U/L*	18.6	24.2	56.5	29.3	26.1	**44**	5–30
**TP**, *g/L*	73.31	66.04	74.09	73.98	71.95	76.9	60–80
**CRTN**, *μmol/L*	72.1	65.5	69.2	45.7	**103.5**	50	60–110
**CysC**, *mg/L*	1.13	0.95	1.02	0.95	**1.26**	0.60	0.62–1.15
**WBC**, *x10^9 cells/L*	7.80	9.01	8.90	9.40	7.90	7.10	4.50–11.00
**RTIC**, *x10^{12} cells/L*	0.115	0.07	0.058	0.059	0.072	0.082	0.02–0.115
**Adverse outcomes**							
**Notable disease** (Organ)	Heart failure (Heart)	Atherosclerosis (Vasculature)	Cerebral infarction (Brain)	Non-insulin diabetes (Metabolic)	Chronic renal failure (Kidney/renal)	Fatty liver (Liver)	
**Time to event**, *years*	+1	+2	+2.5	+3.6	+2	+3.5	
**Other diseases**	-	Fatty liver, Cyst of kidney, Chronic ischaemic heart disease, Diabetes, Obesity, Hypercholesterolaemia	Urinary tract infection, Volume depletion, Hypernatraemia, Hypokalaemia	-	Volume depletion, Acidosis, Hyperkalaemia	Obesity	
**Died** (Circulatory diseases)	No	No	Yes	No	Yes	No	

Continuous variables are represented as Mean±SD. Categorical variables are represented as n (%). Red color indicates an abnormal value that is related to the corresponding adverse outcome. Optimal ranges were obtained from multiple sources in literature. SBP = Systolic blood pressure; DBP = Diastolic blood pressure; PP = Pulse pressure; MAP = Mean arterial pressure; WMH = White matter hyperintensity; GM = Gray matter volume; COp = Cardiac output during pulse wave analysis (PWA); CIp = Cardiac index during PWA; LVEF = Left ventricular ejection fraction; VR = Ventricular rate; BMI = Body mass index; BFM = Body fat mass; HDL = High-density lipoprotein; GLC = Glucose; ALT = Alanine aminotransferase; GGT = Gamma glutamyltransferase; TP = Total protein; CRTN = Creatinine; CysC = Cystatin C; WBC = White blood cells count; RTIC = Reticulocytes count.

Table S5. Characteristics of the external testing dataset, ARIC, compared with the UK Biobank for selected age ranges

**Age range**		**65—90**			**65—70**		
**Datasets**		**ARIC** (n = 5,507)	**UKB** (n = 11,409)	***p* value**	**ARIC** (n = 940)	**UKB** (n = 6,329)	***p* value**
**Female (%)**		3,176 (57.67%)	5,590 (49.00%)	**<0.001**	568 (60.43%)	3,248 (51.32%)	**<0.001**
**Age**		75 [72-80]	70 [68-73]	**<0.001**	69 [68-70]	68 [67-69]	0.305
**SBP**		129 [118-141]	145 [132-159]	**<0.001**	125 [115-136]	143 [131-157]	**<0.001**
**DBP**		66 [59-73]	78 [71-85]	**<0.001**	68 [62-75]	78 [71-86]	**<0.001**
**MAP**		87 [80-95]	101 [93-109]	**<0.001**	87 [80-95]	100 [92-108]	**<0.001**
**Ethnicity**	**White**	4,440 (80.62%)	11,185 (98.04%)		717 (76.28%)	6209 (98.10%)	
	**Black**	1,067 (19.38%)	32 (0.28%)	-	223 (23.72%)	18 (0.28%)	-
	**Other**	NA	192 (1.68%)		NA	102 (1.62%)	

Continuous variables are represented as Median [Q1-Q3]. Categorical variables are represented as n (%). Other ethnicity includes mixed, Caribbean, Chinese, Indian, Bangladeshi, and Pakistani.

Table S6. Characteristics of the *HyperTrajectory* in the overall ARIC cohort.

HyperTrajectory	1 (N = 1,722)	2 (N = 1,132)	3 (N = 806)	4 (N = 961)	5 (N = 726)	6 (N = 160)
HyperScore	0.39±0.20	0.19±0.09	0.35±0.17	0.22±0.13	0.36±0.17	0.08±0.07
**Demographic information**						
Female	782 (45.41%)	916 (80.92%)	406 (50.37%)	685 (71.28%)	275 (37.88%)	112 (70.00%)
Age, *years*	76.04±5.34	75.33±5.04	75.89±5.06	76.46±5.59	75.52±4.96	75.34±5.01
SBP, *mmHg*	129.86±18.16	130.45±18.64	131.38±18.04	130.98±18.68	129.59±17.82	130.11±17.81
DBP, *mmHg*	66.26±10.71	66.64±10.47	67.30±10.89	66.44±10.99	66.56±10.57	66.37±10.80
PP, *mmHg*	63.60±14.79	63.81±15.32	64.07±14.35	64.54±15.07	63.03±14.24	63.74±14.37
MAP, *mmHg*	87.46±11.74	87.91±11.70	88.66±11.91	87.96±12.10	87.57±11.63	87.62±11.73
Current smoker	96 (5.57%)	60 (5.30%)	40 (4.96%)	53 (5.52%)	44 (6.06%)	9 (5.62%)
Diabetic	623 (36.18%)	238 (21.02%)	254 (31.51%)	239 (24.87%)	224 (30.85%)	38 (23.75%)
Heavy alcohol drinker	822 (47.74%)	544 (48.06%)	383 (47.52%)	457 (47.55%)	349 (48.07%)	77 (48.12%)
Hypertension Medication	1,352 (78.51%)	794 (70.14%)	621 (77.05%)	684 (71.18%)	567 (78.1%)	116 (72.5%)
Cholesterol Medication	1,039 (60.34%)	550 (48.59%)	472 (58.56%)	505 (52.55%)	416 (57.30%)	94 (58.75%)
**Imaging/non-imaging measurements**						
WMH, *x10^3 mm^3*	17.77±15.60	13.15±10.53	16.11±15.18	25.21±24.42	20.23±20.3	11.34±8.20
CO, *L/min*	3.62±0.99	3.14±0.79	3.61±1.03	3.25±0.90	3.72±1.08	3.36±0.91
CI, *L/min/m^2*	1.89±0.45	1.77±0.39	1.88±0.47	1.80±0.41	1.93±0.50	1.84±0.41
LVEF, *%*	64.21±7.12	66.28±6.12	64.57±7.04	66.31±6.19	64.24±7.33	66.01±5.74
VR, *bpm*	66.42±9.91	64.81±8.81	66.15±10.09	65.03±9.28	65.24±9.00	65.77±8.81
BMI, *kg/m^2*	29.17±5.64	27.54±5.41	29.79±5.83	27.96±5.61	29.11±5.48	28.18±5.27
BFM, *kg*	28.16±11.45	26.55±10.08	29.66±11.38	26.78±10.76	28.03±10.98	27.3±10.56
Cholesterol, *mmol/L*	4.47±1.07	5.08±1.09	4.56±1.03	4.83±1.10	4.54±1.03	4.73±0.90
LDL, *mmol/L*	2.52±0.89	2.96±0.91	2.59±0.86	2.79±0.92	2.63±0.84	2.64±0.70
HDL, *mmol/L*	1.27±0.33	1.49±0.39	1.27±0.30	1.41±0.36	1.27±0.34	1.46±0.42
Triglycerides, *mmol/L*	1.49±0.79	1.34±0.63	1.54±0.76	1.39±0.70	1.41±0.72	1.36±0.60
HbA1c, *mmol/mol*	42.85±10.55	39.38±6.64	42.22±9.00	40.06±7.82	42.06±9.11	40.03±8.39
GLC, *mmol/L*	6.49±1.76	5.91±1.03	6.42±1.50	6.11±1.29	6.42±1.62	6.08±1.53
CRTN, *µmol/L*	91.76±39.72	81.28±33.27	89.57±29.86	84.21±33.89	93.64±37.86	85.34±43.06
CysC, *mg/L*	1.24±0.48	1.17±0.46	1.24±0.41	1.20±0.43	1.23±0.43	1.20±0.54
WBC, *x10^9 cells/L*	6.05±1.90	5.94±3.63	6.10±2.41	6.00±2.14	5.99±1.79	5.71±1.56
**Adverse outcomes**						
Death (Circulatory diseases)	107 (7.35%)	62 (8.32%)	57 (8.92%)	60 (8.82%)	52 (8.55%)	8 (7.62%)
Cardiac	460 (31.59%)	194 (26.04%)	185 (28.95%)	186 (27.35%)	176 (28.95%)	34 (32.38%)
Brain	142 (9.75%)	79 (10.60%)	48 (7.51%)	70 (10.29%)	60 (9.87%)	11 (10.48%)
Vasculature	70 (4.81%)	29 (3.89%)	26 (4.07%)	29 (4.26%)	30 (4.93%)	1 (0.95%)
Pulmonary	108 (7.42%)	62 (8.32%)	62 (9.70%)	62 (9.12%)	49 (8.06%)	7 (6.67%)
Liver	87 (5.98%)	46 (6.17%)	34 (5.32%)	35 (5.15%)	30 (4.93%)	7 (6.67%)
Kidney	454 (31.18%)	245 (32.89%)	208 (32.55%)	210 (30.88%)	189 (31.09%)	36 (34.29%)
Metabolic	28 (1.92%)	28 (3.76%)	19 (2.97%)	28 (4.12%)	22 (3.62%)	1 (0.95%)
Overall	1,456 (84.55%)	745 (65.81%)	639 (79.28%)	680 (70.76%)	608 (83.75%)	105 (65.62%)

Continuous variables are represented as Mean±SD. Categorical variables are represented as n (%). The percentage of organ-specific outcomes is based on the overall number of outcomes. The percentage of the overall outcomes is based on the total number of participants. SBP = Systolic blood pressure; DBP = Diastolic blood pressure; PP = Pulse pressure; MAP = Mean arterial pressure; WMH = White matter hyperintensity; GM = Gray matter volume; CO = Cardiac output; CIp = Cardiac index; COp = Cardiac output during pulse wave analysis (PWA);

CIp = Cardiac index during PWA; LVEF = Left ventricular ejection fraction; VR = Ventricular rate; BMI = Body mass index; BFM = Body fat mass; LDL = Low-density lipoprotein; HDL = High-density lipoprotein; HbA1c = Glycated hemoglobin; GLC = Glucose; ALT = Alanine aminotransferase; GGT = Gamma glutamyltransferase; TP = Total protein; CRTN = Creatinine; CysC = Cystatin C; WBC = White blood cells count; RTIC = Reticulocytes count.

Table S7. Characteristics of the *HyperTrajectory* in the advanced disease state (top 5% *HyperScores*) in the ARIC cohort.

HyperTrajectory	1 (N = 87)	2 (N = 57)	3 (N = 41)	4 (N = 49)	5 (N = 37)	6 (N = 8)
**HyperScore**	0.78±0.06	0.40±0.06	0.76±0.11	0.54±0.06	0.74±0.07	0.28±0.05
**Demographic information**						
**Female**	44 (50.57%)	40 (70.18%)	6 (14.63%)	39 (79.59%)	14 (37.84%)	4 (50.00%)
**Age, *years***	77.77±5.76	77.42±4.42	77.71±4.48	77.90±6.42	77.57±4.77	76.25±4.03
**SBP, *mmHg***	131.76±19.19	125.07±14.51	130.12±18.37	132.98±20.64	138.68±20.09	125.00±9.47
**DBP, *mmHg***	66.77±9.85	63.53±7.42	66.85±7.90	65.63±8.76	68.97±10.12	66.75±10.54
**PP, *mmHg***	64.99±16.64	61.54±11.50	63.27±16.27	67.35±17.32	69.70±15.82	58.25±7.72
**MAP, *mmHg***	88.43±11.22	84.04±8.80	87.94±9.76	88.08±11.25	92.21±12.13	86.17±9.52
**Current smoker**	4 (4.60%)	6 (10.53%)	4 (9.76%)	3 (6.12%)	6 (16.22%)	1 (12.5%)
**Diabetic**	36 (41.38%)	11 (19.3%)	22 (53.66%)	11 (22.45%)	11 (29.73%)	1 (12.5%)
**Heavy alcohol drinker**	42 (48.28%)	32 (56.14%)	23 (56.10%)	19 (38.78%)	21 (56.76%)	6 (75.00%)
**Hypertension Medication**	74 (85.06%)	42 (73.68%)	32 (78.05%)	40 (81.63%)	29 (78.38%)	6 (75.00%)
**Cholesterol Medication**	69 (79.31%)	31 (54.39%)	31 (75.61%)	24 (48.98%)	23 (62.16%)	6 (75.00%)
**Imaging/non-imaging measurements**						
**WMH, *$x10^3$ mm^3***	32.13±24.37	14.86±13.44	45.58±39.92	78.76±18.12	81.13±28.86	16.69±8.28
**CO, *L/min***	3.41±1.03	3.12±0.92	3.80±1.17	3.29±0.91	3.46±0.89	3.87±1.03
**CI, *$L/min/m^2$***	1.81±0.50	1.75±0.43	1.88±0.50	1.76±0.39	1.85±0.39	2.01±0.41
**LVEF, *%***	62.62±8.44	67.22±5.05	62.33±7.00	65.72±5.52	63.13±7.11	61.62±7.14
**VR, *bpm***	68.98±10.72	62.58±7.98	64.80±9.14	65.12±8.25	65.46±7.97	67.00±6.09
**BMI, *kg/m^2***	30.12±6.05	26.69±5.61	30.14±4.52	31.57±8.80	28.02±3.37	30.42±6.98
**BFM, *kg***	29.39±12.41	23.74±8.90	30.15±11.64	33.77±16.45	26.16±6.97	30.35±13.47
**Cholesterol, *mmol/L***	4.31±0.95	4.76±0.95	4.08±0.93	4.74±0.98	4.53±1.27	4.56±0.73
**LDL, *mmol/L***	2.28±0.78	2.64±0.79	2.27±0.73	2.70±0.78	2.58±0.98	2.76±0.61
**HDL, *mmol/L***	1.21±0.28	1.56±0.53	1.11±0.26	1.40±0.38	1.29±0.33	1.13±0.20
**Triglycerides, *mmol/L***	1.79±1.00	1.20±0.42	1.54±0.69	1.38±0.56	1.43±0.65	1.45±0.36
**HbA1c, *mmol/mol***	44.66±10.36	38.64±5.62	44.20±11.18	40.09±6.57	42.11±10.65	37.71±3.41
**GLC, *mmol/L***	7.05±2.57	5.89±0.97	6.44±1.18	6.25±1.13	6.57±1.49	5.86±0.59
**CRTN, *μmol/L***	87.20±25.36	87.19±25.97	104.20±50.07	72.33±16.88	97.62±32.14	73.04±14.28
**CysC, *mg/L***	1.24±0.29	1.28±0.40	1.36±0.62	1.21±0.30	1.25±0.38	1.08±0.15
**WBC, *$x10^9$ cells/L***	6.54±1.75	5.96±2.04	6.50±1.56	6.62±4.46	5.99±1.50	5.59±1.14
**Adverse outcomes**						
**Death** (Circulatory diseases)	9 (10.59%)	2 (4.44%)	4 (10.81%)	5 (12.20%)	**5 (13.89%)**	0 (0.00%)
**Cardiac**	**27 (31.76%)**	7 (15.56%)	6 (16.22%)	9 (21.95%)	9 (25.00%)	2 (40.00%)
**Brain**	11 (12.94%)	6 (13.33%)	2 (5.41%)	**8 (19.51%)**	4 (11.11%)	0 (0.00%)
**Vasculature**	5 (5.88%)	1 (2.22%)	3 (8.11%)	1 (2.44%)	**4 (11.11%)**	0 (0.00%)
**Pulmonary**	6 (7.06%)	6 (13.33%)	**6 (16.22%)**	3 (7.32%)	1 (2.78%)	1 (20.00%)
**Liver**	2 (2.35%)	1 (2.22%)	1 (2.70%)	**3 (7.32%)**	1 (2.78%)	0 (0.00%)
**Kidney**	25 (29.41%)	**18 (40.00%)**	13 (35.14%)	11 (26.83%)	10 (27.78%)	2 (40.00%)
**Metabolic**	0 (0.00%)	**4 (8.89%)**	2 (5.41%)	1 (2.44%)	2 (5.56%)	0 (0.00%)
**Overall**	**85 (97.70%)**	45 (78.95%)	37 (90.24%)	41 (83.67%)	36 (97.30%)	5 (62.50%)

Continuous variables are represented as Mean±SD. Categorical variables are represented as n (%). The percentage of organ-specific outcomes is based on the overall number of outcomes. The percentage of the overall outcomes is based on the total number of participants. Bold indicates the trajectory with highest number of outcomes if enough outcomes occur. SBP = Systolic blood pressure; DBP = Diastolic blood pressure; PP = Pulse pressure; MAP = Mean arterial pressure; WMH = White matter hyperintensity; GM = Gray matter volume; CO =

Cardiac output; CIp = Cardiac index; COp = Cardiac output during pulse wave analysis (PWA); CIp = Cardiac index during PWA; LVEF = Left ventricular ejection fraction; VR = Ventricular rate; BMI = Body mass index; BFM = Body fat mass; LDL = Low-density lipoprotein; HDL = High-density lipoprotein; HbA1c = Glycated hemoglobin; GLC = Glucose; ALT = Alanine aminotransferase; GGT = Gamma glutamyltransferase; TP = Total protein; CRTN = Creatinine; CysC = Cystatin C; WBC = White blood cells count; RTIC = Reticulocytes count.

Table S8. Sensitivity analysis of the *HyperScore* and *HyperTrajectory* after excluding selected variables, cardiac MR, and brain MR during model development.

	**Excluding selected variables**				**Excluding modalities**	
	LVEF	WMH	Creatinine	All three	Cardiac MR	Brain MR
***HyperScore* RMSE**	0.061	0.088	0.087	0.093	0.097	0.144
***HyperTrajectory* JS distance**	0.269	0.308	0.283	0.273	0.264	0.340

LVEF = Left ventricular ejection fraction; WMH = White matter hyperintensity; MR = Magnetic resonance; RMSE = Root mean square error; JS = Jensen-Shannon

Table S9. Results of fitting a multivariable Cox model with HyperScore in comparison with blood pressure, adjusted for age and sex.

	HR per SD	*p* value	Calibration correlation	Calibration intercept	Calibration slope	NRI (%)	IDI (%)	C-statistic (score)	C-statistic (age+sex)	C-statistic (score+age+sex)	ΔC
**Circulatory diseases and death**											
**HyperScore**	1.247 (1.171–1.329)	**<0.001**	0.993	-0.032	0.987	18.153	0.408	0.691	0.678	0.702	+0.024
**SBP**	1.072 (1.009–1.138)	**0.024**	0.990	-0.048	0.984	7.778	0.012	0.574		0.679	+0.001
**DBP**	0.991 (0.934–1.051)	0.758	0.995	-0.050	0.983	-3.283	0.003	0.512		0.678	0.000
**Brain diseases**											
**HyperScore**	1.408 (1.225–1.619)	**<0.001**	0.942	-0.323	0.930	24.607	0.123	0.636	0.642	0.658	+0.016
**SBP**	1.069 (0.937–1.220)	0.322	0.918	-0.426	0.908	-0.968	0.002	0.561		0.644	+0.002
**DBP**	1.049 (0.921–1.195)	0.473	0.927	-0.473	0.898	7.250	0.001	0.515		0.643	+0.001
**Death**											
**HyperScore**	1.419 (1.163–1.731)	**<0.001**	0.982	-0.183	0.964	34.454	0.098	0.694	0.745	0.755	+0.010
**SBP**	1.010 (0.831–1.228)	0.919	0.961	-0.305	0.941	-1.547	-0.001	0.576		0.745	0.000
**DBP**	1.038 (0.857–1.257)	0.705	0.954	-0.256	0.951	-0.238	-0.001	0.514		0.746	+0.001

SBP = Systolic blood pressure; DBP = Diastolic blood pressure; HR = Hazard ratio; SD = standard deviation; NRI = Net reclassification index; IRI = Integrated discrimination improvement (IDI); C-statistic = Concordance statistic

Table S10. Results of fitting a multivariable Cox model with HyperScore in comparison with other scoring metrics, adjusted for age and sex.

	HR per SD	*p* value	Calibration correlation	Calibration intercept	Calibration slope	NRI (%)	IDI (%)	C-statistic (score only)	C-statistic (age+sex)	C-statistic (score+age+sex)	ΔC
**Circulatory diseases and death**											
**HyperScore**	1.247 (1.171–1.329)	**<0.001**	0.993	-0.030	0.987	18.153	0.408	0.691	0.678	0.702	+0.024
**ACC/AHA**	1.525 (1.415–1.643)		0.982	-0.032	0.990	11.840	0.645	0.702		0.699	+0.021
**Framingham**	1.410 (1.320–1.506)		0.980	-0.034	0.989	10.873	0.560	0.694		0.698	+0.019
**MESA**	1.943 (1.824–2.070)		0.971	-0.025	0.992	32.462	2.570	0.745		0.747	+0.069
**QRISK3**	2.035 (1.941–2.133)		0.993	0.110	1.035	45.911	4.116	0.760		0.762	+0.083
**Carotid IMT**	1.127 (1.064–1.193)		0.993	-0.049	0.984	9.739	0.082	0.589		0.681	+0.002
**Brain diseases**											
**HyperScore**	1.408 (1.225–1.619)	**<0.001**	0.942	-0.323	0.930	24.607	0.123	0.636	0.642	0.658	+0.016
**ACC/AHA**	1.195 (0.993–1.438)	0.060	0.941	-0.388	0.917	3.190	0.011	0.642		0.648	+0.006
**Framingham**	1.092 (0.928–1.286)	0.290	0.946	-0.463	0.901	8.607	0.001	0.609		0.645	+0.003
**MESA**	1.129 (0.958–1.331)	0.149	0.944	-0.467	0.899	9.488	0.004	0.634		0.647	+0.004
**QRISK3**	1.553 (1.360–1.773)	**<0.001**	0.967	-0.234	0.949	25.317	0.178	0.682		0.680	+0.038
**Carotid IMT**	1.215 (1.071–1.378)	**0.003**	0.938	-0.401	0.913	20.648	0.035	0.605		0.654	+0.012
**Death**											
**HyperScore**	1.419 (1.163–1.731)	**<0.001**	0.982	-0.183	0.964	34.454	0.098	0.694	0.745	0.755	+0.010
**ACC/AHA**	1.179 (0.911–1.526)	0.210	0.985	-0.253	0.951	-9.944	0.008	0.735		0.748	+0.003
**Framingham**	1.176 (0.951–1.456)	0.135	0.972	-0.327	0.937	-2.453	0.009	0.697		0.749	+0.004
**MESA**	1.207 (0.974–1.496)	0.086	0.970	-0.245	0.953	-8.171	0.018	0.722		0.751	+0.006
**QRISK3**	1.531 (1.269–1.848)	**<0.001**	0.979	-0.133	0.974	22.910	0.123	0.769		0.766	+0.021
**Carotid IMT**	1.214 (1.013–1.455)	**0.036**	0.966	-0.309	0.940	10.442	0.028	0.632		0.752	+0.007

IMT = Intima-media thickness; HR = Hazard ratio; SD = Standard deviation; NRI = Net reclassification index; IRI = Integrated discrimination improvement (IDI); C-statistic = Concordance statistic

Datasets Variables

Excel file S1. A list of all variables used as input to train the model using the UK Biobank and to externally validate it using ARIC.
